# Mentalization-based treatment versus bona fide treatment for patients with borderline personality disorder in Germany (MAGNET): study protocol of a prospective, multi-centre randomized controlled trial

**DOI:** 10.1186/s12888-025-06809-0

**Published:** 2025-04-11

**Authors:** Sophie Hauschild, Svenja Taubner, Thorsten Vidalón Blachowiak, Ulrike Dinger, Harald Gündel, Sabine C. Herpertz, Jörg Rademacher, Bernhard Strauss, Timo Storck, Ralitsa Vassileva, Ina Burghaus, Manuel Herbst, Simiao Chen, Christoph Nikendei, Sebastian Euler, Jana Volkert

**Affiliations:** 1 Institute for Psychosocial Prevention, University Hospital Heidelberg, University of Heidelberg, Heidelberg, Germany; 2 Department of Psychosomatic Medicine and Psychotherapy, Medical Faculty, Heinrich-Heine University Düsseldorf, Düsseldorf, Germany; 3 Clinic for Psychosomatic Medicine and Psychotherapy, University Hospital Ulm, University of Ulm, Ulm, Germany; 4 Department of General Psychiatry, University Hospital Heidelberg, University of Heidelberg, Heidelberg, Germany; 5 Institute for Psychosocial Medicine, Psychotherapy and Psychooncology, University Hospital Jena, Jena, Germany; 6 Department of Clinical Psychology and Psychotherapy, Psychologische Hochschule Berlin, Berlin, Germany; 7 Coordination Centre for Clinical Trials (KKS), Medical Faculty, University Hospital Heidelberg, Heidelberg, Germany; 8 Heidelberg Institute of Global Health, University of Heidelberg, Heidelberg, Germany; 9 Department for General Internal Medicine and Psychosomatics, University Hospital Heidelberg, University of Heidelberg, Heidelberg, Germany; 10 DZPG (German Center for Mental Health), Heidelberg, Ulm, Germany; 11 Department of Consultation-Liaison Psychiatry and Psychosomatic Medicine, University Hospital Zurich, Zurich, Switzerland

**Keywords:** Borderline Personality Disorder, Mentalization-based therapy, Randomized controlled trial, Health economy, Crisis events, Ecological Momentary Assessment, Mechanisms of change

## Abstract

**Background:**

Borderline Personality Disorder (BPD) is a serious mental disorder. Mentalization-based treatment (MBT) is an evidence-based treatment for individuals with BPD. Specifically, MBT has previously been highlighted for its effectiveness regarding the reduction of suicidal and non-suicidal self-injury (NSSI). Yet, randomized-controlled trials (RCT) on MBT in outpatient settings compared with bona fide treatment (BFT) are still scarce and none has been conducted in Germany. The primary objective of this RCT is to investigate whether outpatient MBT is more effective in the reduction of crisis events (incidences of NSSI and suicide attempts) compared with BFT (namely psychodynamic or cognitive-behavioural psychotherapy) in Germany. Secondary, MBT’s efficacy will be investigated with regard to cost-effectiveness within the German health care system, general and interpersonal functioning, BPD and general symptom severity, social adjustment, quality of life, reduction in psychotropic medication and therapy retention. Additionally, moderator as well as common and treatment specific mediator variables will be investigated.

**Methods:**

Across 5 study sites in Germany, 304 individuals of all genders from age 18 to 65 with a BPD diagnosis and NSSI or suicide attempts in the past will be asked to participate in the study for a period of two years. In the first year, patients will receive either MBT or BFT and will take part in continuous scientific assessments. Scientific assessments will continue after therapy completion up to a 12-month follow up. As primary outcome, crisis events will be assessed via ecological momentary assessment (EMA) in one week per month (four weekly assessments) during the first year, and in one week every three months during the second year. Number of crisis events up to 2 years post randomization will be compared between treatment arms using a log-linear regression model following an intention-to-treat approach. Secondary outcomes and mediator variables will be assessed at several timepoints.

**Discussion:**

This study investigates efficacy of MBT as BPD specific treatment in an outpatient setting compared with BFT in Germany. Results of this study can address a treatment gap in the German healthcare system, and inform about health economic aspects of BPD treatment as well as mechanisms of psychotherapeutic change.

**Trial registration:**

NCT06018272, https://www.clinicaltrials.gov/, August 30, 2023.

**Supplementary Information:**

The online version contains supplementary material available at 10.1186/s12888-025-06809-0.

## Background

Borderline Personality Disorder (BPD) is a severe mental health disorder characterized by at least five of the following nine criteria according to DSM- 5, section II (DSM, [1]): unstable relationships, inappropriate anger, frantic effort to avoid abandonment, affective instability, impulsivity, self-harm/suicidality, dysphoria, stress-related paranoid thoughts, and identity disturbance and dissociation. BPD is the only categorical qualifier that will remain in the new personality disorder classification of the ICD- 11 [2]. Point prevalence in community samples is around 2% [3], lifetime prevalence is around 6% [4]. BPD is the most common personality disorder in clinical populations, with prevalence rates of around 10% in outpatient and 15–25% in inpatient settings [5]. BPD is often associated with comorbid DSM-IV axis I and II disorders: approximately 85% of BPD patients have a 12-months diagnosis of at least one axis I and 74% for another axis II disorder [6]. Sixty-nine to 80% of BPD patients engage in suicidal behaviour and 3–10% commit suicide with a 50-fold heightened risk in comparison to the general population [6–8]. Up to 50% of BPD patients show co-occurring conditions of Post-Traumatic-Stress-Disorder (PTSD) which can be captured by a new diagnosis in ICD- 11 termed “complex PTSD” [9]. BPD accounts for 2.2% of all disability adjusted life years (DALYs) (ranking 3rd in mental disorders in women, and 4 th in men) and suicide accounts for 1.0–2.8% of all DALYs [10]. Furthermore, patients with BPD between the ages of 20 and 50 years have a mortality rate on average 2.30 times higher than the normal population and a loss of life years of between 6.8 and 9.3 years [11]. Studies on the economic burden of BPD on society diverge in their exact numbers due to different cost calculations, either pointing out a main relevance of indirect costs (76.3% of total costs of BPD) or equal relevance of indirect and direct costs [12]. Yet, studies concur in their findings that the economic burden of BPD is strikingly high compared with other psychiatric disorders (e.g. depression or alcohol abuse) with the main driver of direct costs being costs for hospital stays, and the main driver of indirect costs being productivity losses due to disability [12].

Mentalization-based treatment (MBT, [13]) has specifically been developed for the treatment of individuals with BPD. MBT is an integrative psychodynamic treatment rooted in attachment theory [14]. It assumes that mentalizing vulnerabilities lie at the core of the disorder. Mentalizing is the imaginative ability to interpret and understand the behaviour of oneself and of others in terms of mental states and develops in early attachment relationships [15]. Empirical research has shown that mentalizing is highly impaired in individuals with BPD especially with regard to emotionally intense relationships [14]. MBT follows distinct therapy rationales and is assumed to help alleviate BPD symptoms through inter alia improving mentalizing and epistemic trust, i.e. the trust in interpersonally transmitted information being relevant, trustworthy and generalizable for the individual [16]. MBT, alongside dialectical behaviour therapy (DBT; [17]), is the most studied psychotherapy for BPD. Five randomized-controlled trials (RCTs) have investigated the efficacy of MBT in BPD in comparison to psychiatric services [18], structured clinical services including supportive psychotherapy [19, 20], specialist BFT [21] and in adolescents with non-suicidal self-injury (NSSI) who mainly fulfilled criteria for BPD [22]. MBT proved to be superior to bona fide treatment (BFT)/clinical management in NSSI, suicide attempts, psychiatric symptoms, and hospitalization [18, 22] as well as core BPD symptoms [22, 23]. One RCT found MBT as effective as a specialist treatment-as-usual (TAU) for the primary outcome, reduction of Borderline symptom severity, with large effect sizes for both groups; however, there were significantly fewer drop-outs in the MBT group [21]. One independent RCT confirmed positive effects for MBT in comparison to supportive therapy for general functioning and BPD symptoms, suggesting that MBT may address core problems in BPD beyond NSSI and suicidality [20]. MBT is the only treatment for which superiority to clinical management was demonstrated in all primary outcome variables as well as a significantly higher level of employment or academic/occupation training eight years after randomization [24]. Findings also demonstrate that MBT shows superiority over TAU for interpersonal problems and general functioning [25]. In sum, MBT has demonstrated reliable improvements for psychiatric symptoms.

Although the most recent Cochrane review lists MBT as evidence-based treatment for BPD and highlights its effectiveness in treating NSSI and suicidality [26], quality of evidence for efficacy of BPD specific treatments is still rated low indicating the need for further studies. Also reviews and meta-analyses conclude that there is strong need for confirmatory trials with high study quality and sufficient sample sizes [8, 25, 27], cost-effectiveness studies [27] and the inclusion of quality of life and preference measures [25]. McLaughlin et al. [28] and Oud et al. [27] also recommend to investigate the effective ingredients in treatments. Furthermore, although MBT has increasingly been clinically implemented in several German inpatient units as well as in outpatient treatment, its efficacy has not yet been studied in Germany.

## Methods/design

### Aim, design and setting

Based on the above-mentioned observations, we designed this cluster-randomized controlled trial (cRCT). It will be the first in Germany to investigate the efficacy of an outpatient MBT in comparison to BFT (here psychodynamic psychotherapy, PT, or cognitive-behavioural psychotherapy, CBT) for patients with BPD. BFT was chosen as a comparator for reasons of ecological validity. The primary objective of the RCT is to investigate whether MBT is more effective in the reduction of crisis events compared to BFT. Secondary, the trial will investigate whether MBT is superior to BFT with regard to cost-effectiveness, general and interpersonal functioning, BPD and psychiatric symptom severity, social adjustment, quality of life, changes in psychotropic medication and therapy retention. We expect that MBT will prove superior to BFT in all primary and secondary endpoints. We expect MBT to be more cost-effective for estimates on the financial burden of patients on society. Finally, while there are theories about and preliminary evidence for mechanisms of change [29], empirical validation of these mechanisms is lacking [30]. Thus, this trial will investigate moderator and mediator variables potentially associated with treatment outcome. Moderator variables are pre-treatment variables that predict treatment outcome in MBT and BFT (e.g. severity of BPD, treatment preferences, gender, trauma, initial level of mentalization and attachment). Mediator variables, i.e. the process of change, will be investigated comparing similarities and differences in change patterns between MBT and BFT. This is done by tracking changes of common and specific treatment factors in response to various stimuli. Common factors tracked are therapeutic alliance from both perspectives (patient and therapist) and both settings (individual and group) [31] as well as therapy related agency [32]. Specific factors are changes in mentalizing [33], epistemic trust [34], emotion regulation [35], attachment [36, 37], social pain (e. g. emotional reactions to rejection) [38], automatic thoughts [39], as well as therapist variables (treatment adherence and competence; [40, 41], and therapist-patient verbal and non-verbal interaction (attunement, [42]; rupture-resolution [43]).

**Fig. 1:**
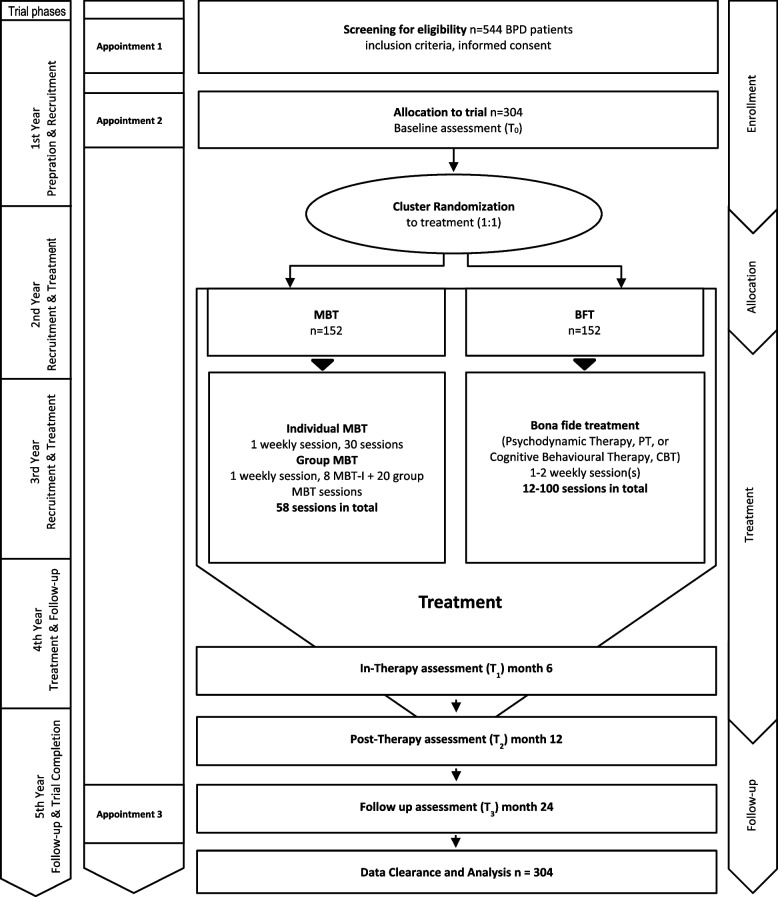
Study flow-chart. Sequence of steps from eligibility assessment to data analysis in both settings

To investigate efficacy of MBT versus BFT, a total of *n* = 544 patients with a BPD diagnosis will be screened for eligibility at five study sites in Germany: University Hospital Heidelberg (Institute for Psychosocial Prevention, ST, and Department of General Psychiatry, SHe), University Hospital Jena (Institute for Psychosocial Medicine, Psychotherapy and Psychooncology, BS), University Hospital Düsseldorf (Clinic for Psychosomatic Medicine and Psychotherapy, UDE & JR), University Hospital Ulm (Clinic for Psychosomatic Medicine and Psychotherapy, HG, JV), Psychologische Hochschule Berlin (Department of Clinical Psychology and Psychotherapy, TS). Of the patients screened, *n* = 304 are expected to fulfil all inclusion criteria including informed consent to participate in the trial, to complete the baseline assessment (T0) and to be allocated to the trial (Fig. 1). Patients will be randomized in groups of 8 (randomization cluster) in a 1:1 ratio to either MBT or BFT using block randomization. Randomization will be stratified by study site. For randomization, study site coordinators will use a centralized web-based tool (randomizer.at) to maintain allocation concealment. Role-based user rights ensure that only approved study personnel may perform randomizations. Main assessments will be conducted for both arms at 6 months (in-therapy assessment, T1), at 12 months (T2) and at 24 months after start of therapy (12-months follow-up, T3). A final sample of *n* = 304 is expected to be obtained for data clearance and analysis using an intent-to-treat protocol. A drop-out of 30% during therapy is expected and acknowledged in our power analysis. Blinding of study team, trial personnel at site as well as patients is not feasible due to the nature of the interventions. Only central assessors will be blind to treatment. Allegiance bias is minimized as the consortium includes representatives of all trial treatments (MBT, group therapy, BFT).

## Participants

Individuals with a BPD diagnosis (ICD- 10: F60.3; DSM- 5: 301.83, ICD- 11: Borderline qualifier) of all genders will be included if they are between 18 and 65 years old and report NSSI or suicide attempts in the past two years as indicated by the International Personality Disorders Examination (IPDE) [44] item “repeated suicidal behaviours, gestures, threats or self-harm”, one of which has to have occurred within the past six months. The informed consent will be obtained in person by the clinicians that carry out the diagnostic assessments. Key exclusion criteria are moderate and severe acute substance use disorder (exception: cannabis use), a diagnosis of schizophrenia or schizotypal personality disorder, bipolar I disorder (DSM- 5), cognitive impairment (IQ < 80 measured with Raven’s progressive matrices (RPM- 2; [45]) in case of suspicion of intellectual disability) or evidence of organic brain disorder, BMI < 16.5, a serious medical condition that will require hospitalization within the next year (e.g., cancer) and no sufficient German language abilities. We do not exclude patients with a broad range of co-morbidities and medication to provide the best possible generalizability to the population of BPD patients in Germany. Patients who require inpatient treatment during psychotherapy, with a stay lasting longer than 4 weeks, are excluded from the study and counted as drop-outs.

### Recruitment strategy

Flyers are sent out to multiplicators. Recruitment in online forums is conducted. Patients contacting the study sites and cooperating institutions will be informed about the study and screened for eligibility if they are interested in participating. The expected completion date of the recruitment period is January 31 st, 2026.

## Interventions

### MBT

Patients in MBT will receive a maximum of 58 sessions in total. Of those, 30 are weekly individual sessions. 28 sessions are weekly group sessions conducted by two therapists, and consisting of 8 introductory sessions of group psychoeducation (MBT-I) followed by 20 group therapy sessions. The duration of MBT is 12 months. The therapists, who must be specialized in PT and at least be in advanced psychotherapeutic training, will receive a 3-day training in MBT as well as an online course and supervision. MBT is manualized and relies on validating the emotional experience of patients that aims to promote mentalizing. The proposed mechanism of change in MBT is to stabilize mentalizing in certain focus areas in order to create a psychic buffer between affect and behaviour to foster affect regulation, reduce impulsivity and promote functional, supportive relationships.

### BFT

Patients in the control arm will receive CBT or PT which are standard psychological psychotherapies in Germany paid for by the health insurances. CBT and PT are “bona fide treatments” (BFT) in Germany. Patients in BFT can choose whether they want to receive CBT or PT and will receive one to two weekly sessions conducted by community experts delivered as short-term psychotherapy (≤ 24 sessions) or long-term psychotherapy (> 24 sessions). Under some circumstances, patients can change their therapist. BFT can be delivered as individual, group or a combination of individual and group treatment as stated in the German Psychotherapy Regulations. CBT can be delivered with up to 80 sessions. The therapists delivering CBT or PT in the control group should at least be in advanced therapeutic training and can´t be certified practitioners in DBT, MBT, schema-focused or transference-focused therapy. CBT is defined as an approach that relies on learning and social psychology, requires an analysis of etiological and maintaining causes of the mental disorder (behavioural analysis) and uses techniques such as operant conditioning, cognitive restructuring and learning new forms of self-control. PT according to the German Psychotherapy Regulations can be delivered with up to 100 sessions. PT is defined as an etiological approach that focuses on unconscious psychodynamics and currently active (neurotic) conflicts as well as problems in functioning. PT therapists use clarification, confrontation and interpretation as core techniques and address phenomena of transference, countertransference and resistance to engage the patient in the therapeutic process and explore topics, emotions, and interpersonal patterns that patients would typically avoid dealing with. Elements of BPDspecific therapies are allowed in BFT; however, BFT should not represent regular MBT, DBT, schema therapy or transference-focused treatment practiced by certified practitioners.

## Additional treatments

Additional treatments except concomitant psychotherapy (including inpatient psychotherapy > 4 weeks) will be allowed in the two arms of the trial. Additional treatment can comprise pharmacotherapy, rescue medication, self-help groups, managed care, crisis interventions in psychiatry. Most trials in BPD so far have allowed concurrent psychotropic treatments [25] which reflects the use of pharmacotherapy in BPD [46]. All additional treatments will be systematically assessed and controlled for in data analysis. Changes in psychotropic treatment are measured as a secondary outcome.

## Procedure

### For an overview of assessments, schedule of enrolment and interventions see Table 1.

Diagnostic assessments to ensure inclusion/exclusion criteria will be carried out by trained clinicians using the Clinical Version of the Structured Clinical Interview for DSM- 5 [47] and the BPD and schizotypal personality part of the IPDE [44]. Raven’s progressive matrices (RPM- 2 [45]) will be used to assess IQ in case the clinicians suspect intellectual disability. Diagnostic assessments will be videotaped and 10% will be selected to be rated by all raters for reliability testing.

**Table 1:**
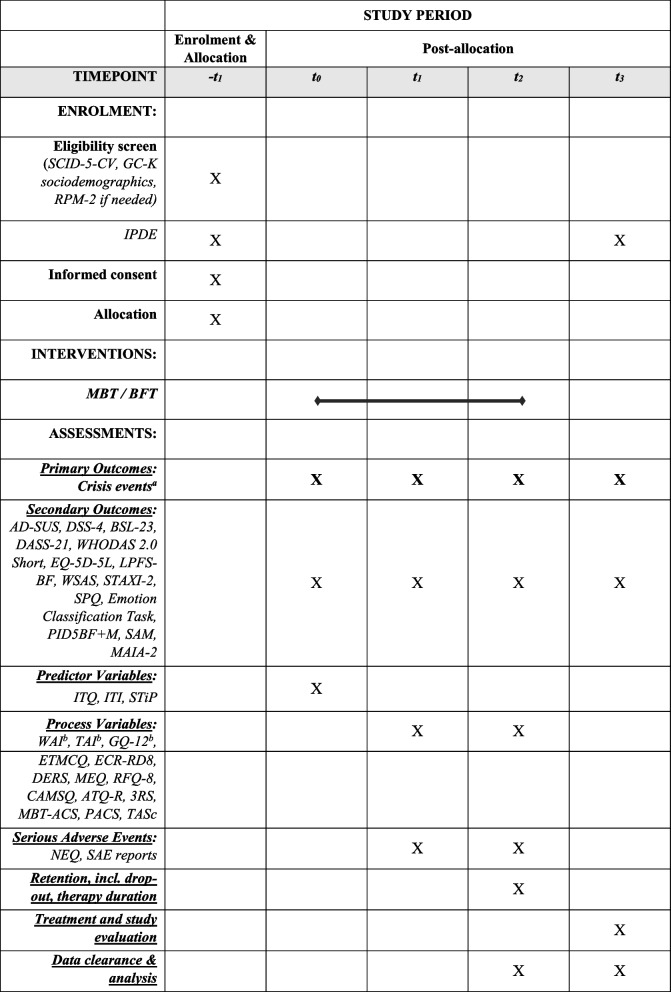
*S*chedule of enrolment, interventions, and assessments

^a^EMA: every second day, one week per month in the first year and one week every three months in the second year

^b^after each therapy session and at t_1_ and t_2_

Data will be gathered and managed via redcap by the data coordinator (SHa) of the PI’s (ST) study site. Clinical monitoring will be conducted by the independent clinical monitor KKS to ensure patient safety, protocol adherence, and data consistency. For important protocol modifications, approval of the ethics committee will be obtained and the protocol published on the clinicaltrials.gov website will be updated.

Treatment duration in BFT is delivered as usual (according to the German Psychotherapy-Guidelines “Richtlinienpsychotherapie”). Thus, it can be shorter or longer than MBT (12 to 100 h); however, the average number of BFT treatment is comparable with 48 sessions per patient [48]. The length of BFT will not be controlled for by the study to ensure BFT conditions as in the German routine health care. Exact treatment length and dose will be assessed. To avoid the effects of attention bias, supervision will be regularly offered to therapists in both arms in their respective methods in a group setting. All interventions are implemented as face-to-face interventions. Individual and group therapists can be the same or different therapists. All therapies in the trial will be paid for by the German health insurances.

All therapy sessions will be video-recorded or, if video is not possible, audio-taped. Ten percent of random sample recordings from early and late phases of therapy across the whole recruitment phase will be analysed by external coders for therapist adherence. Trial results will be communicated by publication in journal articles and by congress participation.

## Instruments

### Eligibility screen

#### International personality disorder examination – borderline and Schizotypal personality disorder sections

The International Personality Disorder Examination (IPDE; [44], German version by [49]) is a clinical interview that follows a semi-structured format, utilized to assess personality disorders according to the classification systems outlined in the ICD- 10 and DSM-IV. For this study, the 15 items regarding BPD, as outlined in the DSM-IV, which are answered on three-point Likert scales (0 = “absent”; 2 = “pathological or meets criterion”) will be used. A time criterion will be added to identify whether the suicidal behaviour and self-harm occurred in the last 24 months and at least one in the last six months. In addition, the IPDE will be used to diagnose schizotypal personality disorder (9 items), as this is an exclusion criterion. The interrater-reliability for BPD diagnosis according to ICD- 10 is between *k* = 0.76 and *k* = 0.78 with a temporal stability of *k* = 0.62 to *k* = 0.65 [50].

#### Structured clinical interview for DSM- 5 – clinical Version

Major mental disorders will be assessed with the German version of the Clinical Version of the Structured Clinical Interview for DSM- 5 disorders (SCID- 5-CV; [47]). The SCID- 5-CV is the latest version of a well-established interview used to assess major mental disorders. The criteria are rated either as yes, fulfilled (or “ + ”), or no, not fulfilled (or “- “).The diagnostic sensitivity/specificity are above 0.70, the kappa coefficient values have been shown to be *k* > 0.70 for almost all diagnoses [51].

#### Raven’s progressive matrices 2, clinical edition

The Raven’s progressive matrices (RPM- 2; [45]) evaluate general cognitive ability, also known as *eductive* or meaning-making ability. In this study, the paper–pencil version of the Standard Progressive Matrices (SPM) will be employed in case of suspected mental retardation. The SPM include five sets of items that show a sequence of incomplete geometrical designs with increasing difficulty. Test-takers are required to identify the correct component to fill in the missing part of the designs from a list of given options. The items in each set follow a different logic, with the sets themselves becoming progressively more challenging. The reliability of the instrument is *r* = 0.75 and *r* = 0.93, depending on the age group [45].

#### Sociodemographics

Age, gender and a proxy for crystalline intelligence will be assessed as sociodemographic variables during the appointment for recruitment and confirmation of eligibility for the patients. To approximate the assessment of crystalline intelligence, the Berlin Test for the Assessment of Fluid and Crystallized Intelligence—Short Form Crystallized Intelligence (BEFKI GC-K) will be used. The BEFKI GC-K [52] is a measure for screening declarative knowledge via 12 questions with four response options each and an overall task duration of 5 min. Reliability of the 12-item scale is *α* = 0.70.

### Primary outcome

#### Crisis events – composite outcome

The primary outcome of the study is the incidence of crisis events as indicated by 1) suicides, 2) suicide attempts, or 3) self-harm behavior.

To assess suicide attempts and self-harm behavior, the following four questions taken from the Self-Harm Behaviour Questionnaire ([53], German version: [54]) will be used: “Have you ever intentionally hurt yourself or caused yourself pain? (e.g. scratched with fingernails or with other sharp objects?)”, “How often have you intentionally hurt or caused yourself pain?”, “Have you ever tried to take your own life?” and “How often did you try to take your own life?”. These questions will be adapted for the Ecological Momentary Assessment (e.g., “Since the last signal have you ever intentionally hurt or caused yourself pain? (e.g., scratched with fingernails or with other sharp objects?”). Only if any of the crisis events occurred, the patient will be asked to indicate the frequency of the event(s).

Crisis events like suicides cannot be reported by the patient themselves but should be taken into account as well (see e.g. Bateman and Fonagy [18]). Suicides are recorded via the Serious Adverse Events (SAE) reports.

To increase ecological validity, the incidence of crisis events 1) – 4) will be assessed during one week each month over the course of 12 months of treatment, and one week every three months post treatment up to the 12-month-follow up (T3). During the assessment week, patients will be asked to complete one online-assessment every other day, resulting in 4 assessments per assessment week.

### Secondary outcomes

#### Crisis events—individually

To gain deeper insights, the incidences of 1) suicides, 2) suicide attempts, and 3) self-harm behavior will be analyzed individually.

#### International personality disorder examination – borderline personality disorder section

In addition to its use for eligibility screening, the IPDE BPD section [49] will be used as a secondary outcome to assess changes in BPD diagnosis.

#### Borderline symptom list – short version

The Borderline Symptom List – Short Version (BSL- 23; [55]) will be used to assess borderline-specific clinical symptoms in the last seven days, i.e., the degree of severity. The items are answered on a 5-point Likert scale ranging from 0 = “not at all” to 4 = “very strong”. The degree of severity is calculated as a mean of the valid items. The internal consistency of the instrument is excellent (*α* = 0.94–0.97) [55].

#### Depression, anxiety and stress scale – 21 items

The self-report Depression, Anxiety and Stress Scale – 21 Items (DASS- 21; [56], German version: [57]) will be used to assess emotional states of depression, anxiety and stress in the last seven days. Each of the three scales consists of seven items which are answered on a 4-point Likert scale (0 = “did not apply to me at all”; 3 = “applied to me very much or most of the time”). The score for each scale is quantified by adding up the scores of the items. The reliability of the scales is between *α* = 0.78 and *α* = 0.91 [57].

#### World health organization disability assessment schedule 2.0 short form

The World Health Organization Disability Assessment Schedule 2.0 Short Form (WHODAS 2.0 short; [58]) is a self-report tool for evaluating health and disability in the last 30 days that covers the following domains of functioning: cognition, mobility, self-care, getting along with others, life activities and participation in community activities. The 12 items are answered on a 5-point Likert scale (1 = “none”; 5 = “extreme”). A total score is obtained as a sum of all items. The reliability of the scale tested in a sample of outpatients with psychotic disorders is good (*α* = 0.89) [59] and has showed a good to excellent internal consistency in a sample of patients with anxiety and stress disorders [60].

#### EQ- 5D- 5L

The EQ- 5D- 5L [61, 62] is a self-report, generic questionnaire that contains two parts: a five-item descriptive system and a visual analogue scale resembling a thermometer, with a range of 0 to 100. It measures five dimensions: mobility, self-care, usual activities, pain/discomfort, and anxiety/depression. Respondents can choose from five options: level 1 (no problems), level 2 (slight problems), level 3 (moderate problems), level 4 (severe problems), and level 5 (extreme problems or'unable to'). The ratings are added up to create a health profile. The reliability of the scale in patients with depression has been shown to be *α* = 0.77 [63].

#### Adult service use schedule

The Adult Service Use Schedule (AD-SUS; [64]) will be adapted for BPD and used to assess employment status, gross pay per year as well as hospital services, other BPD-associated services and community services used, use and dosage of (psychotropic) medication and indirect costs associated with BPD. The questions refer to the last 6 months. The original AD-SUS instrument is used as an interview, but for this study it will be adapted to a self-report questionnaire.

#### Dissociation-Tension scale- 4

The Dissociation-Tension Scale- 4 (DSS- 4, [65]) is a four-item self-report questionnaire used to assess acute dissociative symptoms. The items are answered on a 10-point Likert scale (0 = “not present” to 9 = “very strong”). The total dissociation score is calculated by forming the mean of the four items. The internal consistency of the scale is good (*α* = 0.87) [65].

#### Work and social adjustment scale

The Work and Social Adjustment Scale (WSAS; [66, 67], German version: [68]) is a measurement tool that allows individuals to self-report their level of functional impairment caused by a specific problem. It consists of five items: work, home management, social leisure, private leisure, and relationships, which are answered from 0 (“not at all impaired”) to 8 (“very severely impaired”) and can be summed up to provide an overall score, ranging from 0 to 40. A score exceeding 20 suggests moderately severe or more severe impairment, while scores between 10 and 20 indicate significant functional limitations. Scores below 10 are categorized as subclinical. The internal consistency of the instrument is good (α = 0.89) [68].

#### Levels of personality functioning scale- brief form

Assessment of the level of personality functioning will be done with the 12-item Level of Personality Functioning Scale – Brief Form 2.0 (LPFS-BF; [69], German version by Spitzer et al. [70]). This self-report questionnaire assesses levels of personality functioning according to Section III of the DSM–5. It comprises 12 facets that are organized into four subscales: Identity, Self-Direction, Empathy, and Intimacy. The scores for the subscales are quantified by the sum of the items, which are answered on a 4-point Likert scale ranging from 1 = “not at all true” to 4 = “very true”. The subscales further combine to form two broader domains: Self-Functioning and Interpersonal Functioning (self- and interpersonal pathology). The total scale and both of its subscales exhibit a high internal consistency (*ω* ≥ 0.83) in the German version [70].

#### State-Trait anger expression inventory – 2

The State-Trait Anger Expression Inventory – 2 (STAXI- 2; [71], German version: [72] is a 51-item self-report questionnaire which measures anger experience, expression and control on four-point Likert scales. The instrument is composed of five scales: State-Anger, Trait-Anger, Anger-Control, Anger Expression in and Anger Expression out. In this study the 10-item Trait-Anger scale will be used which assesses an individual´s enduring tendency or disposition to experience feelings of anger as a stable personality-like trait over extended periods of time. It captures the person´s inclination or temperament towards becoming angry. This scale can furthermore be divided into two subscales: angry temperament and angry reaction. The items are answered on a 4-point graded scale from 1 = “completely disagree” to 4 = “completely agree”. The score of the scale is obtained by the sum of its items. Reliability of the Trait-Anger scales of the German version ranges between *α* = 0.79 and *α* = 0.91 [72].

#### Social pain questionnaire

The Social Pain Questionnaire (SPQ; [38]) is a 10-item self-report instrument that assesses emotional reactions to social exclusion, rejection and relational devaluation. Each item is answered on a 5-point Likert scale ranging from 0 = “Applies not at all to me” to 4 = “Applies exactly to me”. The total score is derived by calculating the average of all item scores. The composite reliability of the questionnaire is excellent (0.94) [38].

#### Emotion classification task

In the present study an emotion classification task that has been used in prior studies with patients with BPD [73–75] will be employed to assess changes in threat hypersensitivity. Participants are presented with 80 faces, each unambiguously showing one of four emotional expressions (angry, fearful, happy, neutral). The faces appear for a brief time (150 ms). The goal of the task is to correctly classify these facial expressions. The pictures presented are from the following picture sets: the Karolinska Directed Emotional Faces (KDEF) [76], the NimStim Face Stimulus Set (http://www.macbrain.org/resources.htm), Pictures of facial affect [77], and the FACES database [78]. The task's collected behavioural data encompasses two key measurements: the ratio of accurate responses (specifically, correct emotion classification) and the time it takes to respond in trials where the emotion classification is correct. The task takes roughly 10 min to complete.

#### Personality inventory for DSM- 5 and ICD- 11 – brief form modified

The 36-item self-report Personality Inventory for DSM- 5 and ICD- 11 – Brief Form Plus Modified (PID5BF + M; [79] will be used to assess the following six ICD- 11 and DSM- 5 personality trait domains: negative affect, detachment, antagonism, disinhibition, psychoticism and anankastia. The domains are calculated by forming mean scores of the six items of each domain. Each domain consists of three different facets (e.g. emotional lability, anxiousness and separation insecurity for negative affect) with each facet being measured with two items. The items are answered on 4-point Likert scales (0 = “very untrue or often untrue”; 4 = “very true or often true”. The internal consistency of the scales ranges between *ω* = 0.59 to *ω* = 0.75 [79].

#### Multidimensional assessment of interoceptive awareness

The Multidimensional Assessment of Interoceptive Awareness (MAIA- 2; [80], German version by Eggart et al. [81] is a 37-item self-report questionnaire that measures interoceptive body awareness with eight scales (Noticing, Non-Distracting, Not-Worrying, Attention Regulation, Emotional Awareness, Self-Regulation, Body Listening, Trusting). The items are answered on 6-point Likert scales (0 = “never”; 5 = “always”). Subscale scores are determined by computing the mean of the items within each scale. The internal consistency of the scales ranges from *ω* = 0.70 to *ω* = 0.90, measured on a sample of patients with depression posthospitalization [80].

### Predictor variables

#### International trauma questionnaire

The 18-item International Trauma Questionnaire (ITQ; [82] German version by Lueger-Schuster et al. [83] will be used to assess symptoms of Posttraumatic Stress Disorder (PTSD) and complex PTSD (CPTSD) as defined in ICD- 11. It consists of three PTSD-clusters (re-experiencing, avoidance and sense of threat) and three clusters of disturbances in self-organization (DSO) (affective dysregulation, negative self-concept and disturbances in relationships) with six items each, and functional impairments regarding social life, work, and other important areas of life with three items. The ITQ identifies a respondent's primary traumatic event and participants respond to all questions specifically related to their identified traumatic event. All items are answered on a 5-point Likert scale (0 = “not at all”; 4 = “extremely”). Scores ≥ 2 are indicative of the presence of a symptom. The score of each cluster is calculated by the sum of its items. A final PTSD and DSO score can be computed by summing up the underlying clusters. The DSO scores are indicative of CPTSD. The simultaneous diagnosis of both PTSD and CPTSD is not permitted; an individual can only be diagnosed with one of these conditions. The reliability of all the subscales of the original instrument is *α* ≥ 0.79 [82]. The German version of the ITQ can be considered a valid measure for ICD- 11 CPTSD [84].

#### International trauma interview

The International Trauma Interview (ITI; [85], German version: [86]) is a semi-structured clinical interview used to assess both PTSD and CPTSD in alignment with the diagnostic criteria of the ICD- 11. There are three symptom clusters for PTSD, with two symptoms for each cluster, and three symptom clusters for DSO, also with two symptoms for each. The items are answered on 5-point Likert scales (0 = “not existent” or “not at all”; 4 = “extreme”). Scores of 2 or higher suggest the presence of a symptom. Only a diagnosis of PTSD or CPTSD can be given. The internal consistency measured in a civilian and military sample is satisfactory to good (PTSD: *α* = 0.73 and *α* = 0.83; DSO: *α* = 0.85 and *α* = 0.87; total instrument: *α* = 0.84 and *α* = − 0.86, respectively). The interrater agreement is good (*α* = 0.89) [87].

#### Semi-Structured interview for personality functioning

The semi-structured Interview for Personality Functioning DSM- 5 (STiP 5.1; [88], German version by [89]) will be used to assess the level of personality functioning based on the DSM- 5 alternative model for personality disorders. The interview consists of two domains of personality functioning: Self-functioning (identity and self-direction) and Interpersonal functioning (empathy and intimacy) which are assessed with 28 open questions. The items are answered on 5-point scales ranging from 0 = “little or no impairment” to 4 = “extreme impairment”. The scores of the domains and the overall score are determined by the mean scores of the underlying questions. The interrater-reliability and the internal consistency of the German version are excellent (ICC = 0.93 and *α* = 0.96, respectively) [90].

### Process variables

#### Working alliance inventory – short revised

The Working Alliance Inventory – Short Revised (WAI-SR; [31]; German version by Wilmers et al. [91]) is a self-assessment tool used to assess the current therapeutic relationship. Both, patient and therapist versions will be used. It consists of 12 items that can be categorized into three subscales: attachment, tasks, and goals. Respondents rate each item on a 5-point Likert scale ranging from 1 = “never” to 5 = “always”. The subscale scores and total score are calculated by the means of the underlying items. The reliability of the scales is good (*α* = 0.81–0.91) [91].

#### Therapeutic agency inventory

The Therapeutic Agency Inventory (TAI; [32]) uses 15 items which are rated on a 5-point Likert scale (1 = “not true”; 5 = “very true”) to assess how patients intentionally influence the psychotherapeutic change process. The instrument consists of three subscales: in-session activity (active contribution of the patient during therapy sessions), therapy-related processing (the processing of the patient during sessions), and therapist-oriented passivity (degree of feeling of responsibility and influence of the patient). The score of the subscales and the score of the total scale are operationalized by the mean of its items. The internal consistency for the whole instrument is *α* = 0.84 with a range of *α* = 0.73 and *α* = 0.80 for the factors [32].

#### Group questionnaire – short version

The 12-item self-report Group Questionnaire – Short Version (GQ- 12 [92]) is the short form of the 30-item Group Questionnaire (GQ; [93], German version: GQ-D by Bormann et al., [94]) that enables clinicians to measure on 7-point Likert scales (1 = “not at all true” to 7 = “very true”) helpful aspects of relationships within groups. The questionnaire consists of three subscales: positive bond, positive work and negative relationships which enable to track the group cohesion, quality of work relationships, and negative relationship aspects over the course of group therapy. The items of each subscale are summed to form the total subscale scores. There is no provision of an overall score. The internal consistencies of the three subscales are *α* = 0.77, *α* = 0.86 and *α* = 0.58 [95]. Because the GQ- 12 has not been validated in German, the corresponding German items of the validated GQ-D [94] will be used. For therapists, the items were adapted and the adapted version will be used to assess the therapists’ perspective on the therapeutic bond and work in the group.

#### Self-Assessment manikin

The Self-Assessment Manikin (SAM; [96] is a non-verbal assessment technique that measures pleasure, arousal, and dominance in response to various stimuli. It uses pictorial representations, such as a happy or unhappy figure for pleasure and an excited or relaxed figure for arousal. The dominance dimension is represented by changes in figure size, with a larger figure indicating greater control. SAM allows for a 9-point rating scale for each dimension by allowing the subject to place an “x” between any two figures or on any of the five figures in each scale. The SAM was validated with the Semantic Differential rating system [97], yielding the following correlations for the computerized version of SAM: *r* = 0.97 for pleasure, *r* = 0.94 for arousal and *r* = 0.18 for dominance [96]. Presentation of scales will be adapted for smartphone use.

#### Difficulties in emotion regulation scale

The Difficulties in Emotion Regulation Scale (DERS; [35], German version by Ehring et al., [98]) is a 36-item self-report questionnaire which measures emotion dysregulation. The instrument consists of six factors: Nonacceptance of emotional responses, difficulties engaging in goal-directed behaviour, impulse control difficulties, lack of emotional awareness, limited access to emotion regulation strategies and lack of emotional clarity. Items are rated on an ordinal 5-point scale from 1 (“almost never”, 0–10%) to 5 (“almost always”, 91–100%). Higher scores are indicative of a higher level of impairment in emotion regulation. The total score of the scale ranges from 36 to 180. The internal consistency of the original scales is good (from *α* = 0.80 to *α* = 0.89) [35]. The internal consistency of the instrument has been shown to be higher in clinical samples, with values ranging from *α* = 0.84 to *α* = 0.91 for the subscales and a reliability for the total scale of *α* = 0.95 [99].

#### Certainty about mental states questionnaire

The Certainty About Mental States Questionnaire (CAMSQ; [33]) is a self-report instrument developed both in English and German that measures the level of mentalizing on two scales each containing ten items: Self-Certainty (interpreting the mental states of oneself) and Other-Certainty (interpreting the mental states of others). The self- and other-certainty scores are quantified by calculating the mean of the underlying items. The items are answered on 7-point frequency scales from 1 = “never” to 7 = “always”. Internal consistency is high for both scales (*ω* = 0.88 and *ω* = 0.89 respectively) [33].

#### Mentalizing emotions questionnaire

The 16-item Mentalizing Emotions Questionnaire (MEQ; [100]) aims to capture different facets and levels of competence in mentalizing emotions, and was developed based on the Reflective Functioning Scale [101]. The instrument consists of three factors: Self, Communicating and Other. The scores for each factor are determined by computing the mean of its underlying items, which are answered on 7-point Likert scales ranging from 1 = “never” to 7 = “always”. The Self and Communicating scales of the MEQ will be assessed after individual therapy sessions, the Other-scale after group therapy sessions. The internal consistency was shown to be high for all scales, with α = 0.95 for the overall scale, α = 0.94 for the Self-scale, α = 0.92 for Communicating and α = 0.94 for Other [100].

#### Reflective functioning questionnaire

The German 8-item Reflective Functioning Questionnaire (RFQ- 8; [102]) based on the Reflective Functioning Questionnaire (RFQ; [101]) is a self-report questionnaire used to assess impairments in mentalizing. The items are answered on a 7-point scale from 1 („Do not agree at all “) to 7 („Agree completely “). The instrument consists of two subscales: Certainty and Uncertainty about mental states of others and oneself (Hypo- and Hypermentalizing). The internal consistencies of the original instrument in a clinical sample are *α* = 0.77 and *α* = 0.65 and the test–retest reliability over a period of 3 weeks *rs* = 0.84 and *rs* = 0.75, respectively [101].

#### Epistemic trust, mistrust and credulity questionnaire

The Epistemic Trust, Mistrust and Credulity Questionnaire (ETMCQ; [34], German version by Nolte et al. (under review) [103]; Weiland et al. (submitted) [104]) is a 15-item self-report questionnaire for the indirect assessment of epistemic trust with the three scales Trust, Mistrust and Credulity. The items are answered on a 7-point Likert scale. The reliability of the full scale of the English version lies between *α* = 0.71 and *α* = 0.78 [34].

#### Experiences in close relationships – revised screening version

The Experiences in Close Relationships – Revised Screening Version (ECR-RD8; [36]) is based on the Experiences in Close Relationships – Revised (ECR-R; [105]). It measures attachment avoidance and attachment anxiety concerning partner-related expectations and experiences with four items each, on a 7-point ordinal scale ranging from 1 (“strongly disagree”) to 7 (“strongly agree”). The scores for each scale are obtained by a sum of the four items. Internal consistency is *ω* = 0.87 for the attachment anxiety scale and *ω* = 0.91 for the avoidance scale [36].

#### Automatic thoughts questionnaire – revised

The Automatic Thoughts Questionnaire – Revised (ATQ-R, [106]; German version by [39]) is a questionnaire used to assess the frequency of occurrence of positive and negative automatic thoughts in the last seven days. For this study only the 8-item short version of the scale for negative self-statements will be used. The items are answered on a 5-point Likert scale (1 = “not at all”; 5 = “all the time”). The score of the scale is operationalized by the mean of the items. The internal consistency is *α* = 0.84 in a healthy population and *α* = 0.94 in a clinical population [39].

#### Mentalization-Based treatment adherence and competence scale

The Mentalization-Based Treatment Adherence and Competence Scale (MBT-ACS; [40]) allows evaluators to assess therapist activity and appropriateness in five essential domains of MBT using Likert scales (1 = “not at all”; 7 = “extensively”) and descriptive anchors. The scale assesses the frequency (adherence) and the quality (competence) of therapists'actions. Clinicians whose average scores exceed 3.5 are considered to have reached acceptable levels of adherence and competence. Beforehand, our workgroup carried out an authorized translation of the scale into German.

#### Comparative psychotherapy process scale

The Comparative Psychotherapy Process Scale (CPPS, [41]) is a concise assessment tool designed to evaluate the unique characteristics of psychodynamic-interpersonal (PI) and cognitive-behavioural (CB) treatments by examining therapist activity and techniques employed during therapy sessions. It comprises 20 items which can be rated on a 7-point Likert-type scale ranging from 0 (“not at all characteristic”) to 6 (“extremely characteristic”). It consists of two subscales: the PI subscale measures therapist activities and techniques that are significantly emphasized in PI therapy compared to CB therapy, while the CB subscale assesses techniques and therapist activities that are significantly emphasized in CB therapy rather than PI treatment.

#### Rupture resolution rating system

The Rupture Resolution Rating System (3RS; [43]) is an observational tool used to identify the frequency of alliance ruptures and resolution processes in therapy sessions. The system categorizes ruptures into withdrawal ruptures and confrontation ruptures. It consists of codes for seven markers of withdrawal ruptures, seven markers of confrontation ruptures, and ten resolution strategies, all defined based on observable behaviours of the patient and therapist. Additionally, using 5-point Likert scales ranging from 1 (“no significance”) to 5 (“high significance”) coders rate the clinical impact of overall withdrawal markers, confrontation markers, individual rupture markers, and resolution strategies on the therapeutic alliance throughout the session. The degree of rupture resolution and the extent to which the therapist contributed to or worsened the ruptures are also rated using 5-point Likert scales (1 = “poor resolution”; 5 = “very good resolution”). The interrater reliability is between ICC = 0.85 and ICC = 0.98 [107]).

#### Patient attachment coding system

The Patient Attachment Coding System (PACS; [37]) evaluates clients' attachment during a single therapy session, irrespective of the topics discussed or therapeutic approach. A verbatim transcription of the session is analyzed, and the coder identifies 59 discursive markers as they appear during the clients' speech turns. Each marker plays a distinct role in attunement regulation and can be assigned to a specific word, utterance, or entire speech turn. The coder rates these markers on a scale from 1 to 7, with increments of 0.5, across 12 subscales. The rating reflects both the frequency and intensity of the markers associated with each subscale. These subscales are then summed up to three main scales (proximity seeking, exploring and resistance) which are used to obtain a global score of attachment (balance). Interrater reliability between two raters for the three-way classification is *κ* = 0.87 [37].

#### Therapist attunement scales

The Therapist Attunement Scales (TASc; [42]) will be used to assess therapists' attunement and attachment status. It utilizes verbatim transcriptions of the psychotherapy sessions for the assessment. Each intervention of the therapist is analyzed, one of seven distinct and non-overlapping “form” codes is assigned to it (e.g. inquiry or clarification). If any utterances have been coded, they can be further categorized using one of 40 attunement markers outlined in the manual. These markers are defined by specific content, such as emotions or significant relationships, and form, like expression and clarification. The frequency and intensity of the markers are rated on five 7-point scales which are used to obtain a global score and the classification of the therapist’s attunement [42]. Interrater reliability between two raters is *κ* = 0.80. The correspondence of the TASc with the AAI for three-way classifications is *κ* = 0.81 [108].

### Serious adverse events

In line with the ICH guidelines for expedited reporting of serious adverse events [109], study personnel and/or therapists will report occurrences of serious adverse events to the coordinating investigator (CI) via email or phone call promptly, but at the latest after 7 days. Serious adverse events will be defined according to the above-mentioned guidelines as any untoward medical event in a participating patient resulting in death or life-threat, requiring inpatient hospitalization or prolongation of existing hospitalization, resulting in persistent or significant disability/incapacity, or likely leading to one of these consequences without adequate intervention. Upon initial report of the occurrence, the CI/study staff will send out a report form via email, which the reporting person will submit immediately, but at the latest 7 days after receiving the survey. The report form will entail an assessment of the event itself, of causality, and of treatment phase where the event occurred. The CI evaluates every reported serious adverse event regarding its causality and expectedness according to treatment and study protocol. The CI or a representative forward the information to the DSMB. The DSMB chairperson informs the CI after receipt of the information whether an ad hoc meeting is required. When new information becomes available, a follow-up report will be sent out to the reporter assessing the course of events following including measures taken and their outcome.

#### Negative effects questionnaire

The 20-item Negative Effects Questionnaire (NEQ; [110] will be used to assess the different types of negative effects of psychological treatments. The instrument measures the following five scales: symptoms, quality, dependency, stigma and hopelessness. The items are answered on a 5-point Likert scale from 0 = “not at all” to 4 = “extremely”. The scores of the subscales are obtained by summing up the items. The internal consistency of the original instrument is excellent (*α* = 0.95) with reliabilities of the specific factors ranging from *α* = 0.72 to *α* = 0.93 [110].

Treatment and study evaluation

#### Treatment and study evaluation interview

A semi-structured interview developed based on Krause et al. [111] will be used to assess how patients experienced the therapy and the study, what aspects they perceived as positive or negative, and what impact the therapy had on their symptoms and their life.

### Power calculations and sample size

In an RCT of MBT vs. structured clinical management (SCM), Bateman and Fonagy [19] encountered a mean count of 1.8 cases of critical behaviour in MBT vs. 2.6 cases in the SCM group at 12 months of treatment, resulting in a relative risk of 0.7. In another randomized controlled study of MBT vs. standard psychiatric care [18], the median count of self-mutilation in a 6-months period was reduced from 9 to 1 in MBT and from 8 to 6 in standard psychiatric care. As we expect our comparator to be more effective than the comparators used in the previous studies, we are raising the relative risk in order not to overestimate the effect of MBT. Accordingly, we assume 10 events per patient in the control group and 7.5 cases in the MBT group for the whole 24-months period, and would rate a relative risk of 0.75 [112] as sufficiently relevant requiring a model setting as described below to successfully reject the null hypothesis of no effect. As a more conservative approach, we assume less events (but the same relative risk), i.e. 5 events in the control group per patient and 3.75 in the treatment group. Therefore, a log-linear model (Poisson regression) with treatment as explanatory variable, and number of crisis events in 24 months as response, with a mean of 5 events expected for the BFT and 3.75 for the MBT group, results in a relative risk of 0.75. A test of the null hypothesis “no association between treatment and primary endpoint” at α = 0.05 level rejected with a power of 78% if 8 patients could be enrolled per cluster and 38 clusters could be randomized simulated with R assuming a dropout rate of r = 30% and an intraclass-correlation coefficient of 0.1 (ICC). The dropout rate is taken into account using the correction factor of 1/(1-r)^2 according to [113]. The sample size was corrected with help of the ICC according to [114].

### Compliance/rate of loss to follow-up

Building on similar trials comparing MBT with BFT for BPD patients we expect a compliance rate of about 70%, with Bateman and colleagues [19] achieving 99/134 = 73% compliance over 18 months and Jorgensen and colleagues achieving 63/85 = 74% compliance over 24 months [20]. Both Poisson regression for analyzing the primary outcome and multilevel models for analyzing the secondary outcomes handle unbalanced data with different number of measurements per person [115]. As such, the intent-to-treat sample is used in all analyses instead of complete cases.

### Statistical analysis

All analyses will be specified in a statistical analysis plan.

### Primary analysis

The purpose of this trial is to show whether the (incidence) rate of crisis events (suicides, suicide attempts, self-harm behavior recorded throughout therapy and up to 12 month after therapy) is lower for BPD-patients undergoing MBT than for BPD-patients undergoing a BFT. The null-hypothesis (H01) to be tested can be formulated as follows: The (incidence) rates of crisis events in the MBT and in the BFT group are the same. A log-linear (Poisson) regression model (GLMM) will be used to fit with treatment group, sex and age as explanatory variables, along with log of observation (treatment + follow-up) time as an offset. The randomization cluster will be considered as random effect. Poisson regression lends itself to analysis of count data, considering that more events of suicidal behavior within a time frame are worse than one, and controlling for the period of observation by using it as an offset in the model. The null hypothesis (H01) of no treatment effect will be tested using Wald type tests on the regression coefficient of the treatment group (MBT vs. BFT). Tests will be two-sided at the 0.05 level.

Missing values will be replaced using multiple imputation using fully conditional specification methods (FCS) considering randomization cluster, sex, age and the respective value over time.

### Secondary analyses

The primary analysis will be repeated for 1) suicides, 2) suicide attempts, and 3) self-harm behavior. Number of events and person-time will be tabulated against timepoint and treatment arm.

Further key secondary endpoints are the EQ- 5D- 5L, the WHODAS 2.0 Short, DASS- 21 and the BSL- 23.

The EQ- 5D- 5L, BSL- 23 and the WHODAS 2.0 Short will be analyzed by time point using a linear mixed model with baseline value, treatment group, sex and age as explanatory variables. The randomization cluster will be considered as random effect. Missing values will be replaced using multiple imputation using FCS considering randomization cluster, sex, age and the respective value over time.

The DASS- 21 will be categorized. Ordinal regression will be used for analyses using the baseline category, sex, age, and treatment group as explanatory variables. Analyses will take place by time point. The randomization cluster will be considered as random effect. Missing values will be replaced using multiple imputation using FCS considering randomization cluster, sex, age and the value over time.

## Safety analyses

### (Serious) adverse events (as reported)

Adverse events will be categorized by seriousness criteria, relatedness to study treatment and relatedness to study participation. Number of events, number and proportion of patients with at least one event will be tabulated against treatment arm.

#### NEQ

The scores of the five subscales (symptoms, quality, dependency, stigma and hopelessness) will be tabulated against timepoint (t_1_, t_2_) and treatment arm using standard descriptive measures (Mean, Min, Max) and number of patients with non-missing scores.

For each item, number and proportion of patients will be tabulated against timepoint and treatment arm.

### Cost-effectiveness analysis

A within-trial cost-effectiveness analysis (CEA) will be conducted from a societal perspective. We assess the health-related quality of life using EQ- 5D- 5L [61, 62] to calculate the quality-adjusted life-years (QALYs). The patients will be asked to complete the EQ- 5D- 5L questionnaire at baseline (T0), in-therapy assessment (T1), post-therapy assessment (T2), and follow-up assessment timepoint (T3). The estimated costs for the two treatment groups are composed of direct medical, direct non-medical, and indirect costs. The direct medical costs include the intervention costs and BPD-associated medical costs, which will be calculated by multiplying resources by their corresponding unit costs. Data on the use of health and social services will be collected via a modified version of the Adult Service User Schedule (AD-SUS) adapted for use in the BPD patients through self-report [116]. The baseline AD-SUS will cover the period of 6 months prior. At the T1, T2 and T3 follow-up assessments, the AD-SUS will cover the period since the last assessment. The unit costs will be collected from regularly published sources and will be updated on a yearly basis as there is no obligatory unit cost list in Germany [117, 118]. The intervention costs of MBT or BFT, in contrast, will be collected using the records from the RCT, including activities, facilities, and overhead costs. We also include the costs of MBT or BFT by project staff spent with the patients on the telephone or in person, clinical training, supervision, and materials. Indirect costs will be analyzed using the Human Capital Approach, which measures the productivity losses as a result of time off work due to absenteeism or reduced productivity at work due to work disability.

Both costs and QALYs are discounted at 3% per year. Each treatment modality will calculate the incremental cost-effectiveness ratio (ICER) separately. Then we will perform an incremental analysis to select a cost-effective intervention strategy. Sensitivity analyses will be conducted to assess the robustness of the results depending on protocol deviations.

### Interim analyses

No interim analysis to stop early for futility or efficacy is planned.

However, regular interim safety analyses are planned to ensure the safety of the trial participants. For that, an independent DSMB will be set up to make recommendations for the continuation or, if necessary, termination of the study in the event of safety risks.

## Discussion

This study investigates the efficacy of MBT for treatment of individuals with BPD in an outpatient setting compared with bona fide treatment in Germany. MBT has previously been proven effective for treating individuals with BPD [18, 19, 24], and has been specifically highlighted for its effectiveness in reducing suicidality and non-suicidal self-injury [26]. The overarching goal of the trial is to reduce the burden of disease for BPD in Germany by investigating whether implementing MBT as a BPD-specific treatment within the German health-care system might be beneficial and cost-effective. As the intervention addresses a severe challenge in the German health care system, namely reducing crisis events in individuals with BPD, the trial targets a main cause of individual pain and societal costs associated with productivity losses and need for crisis interventions associated with the disorder. Frequent crisis events may inter alia underlie high prevalence rates of individuals with BPD in inpatient settings [5] as options to treat individuals with acute crises adequately within outpatient settings are still limited in Germany. As an outpatient treatment combining group and individual sessions, MBT offers an intensive treatment without the extensive costs associated with hospital stays. Results of this study can thus address a treatment gap in the German healthcare system. Because MBT is not a common treatment in Germany and psychological treatments are rarely conducted in a combination of individual and group treatment, the training of study therapists and implementation of the group component will be a practical challenge for the RCT.

In this study, the control group will receive an evidence-based treatment (CBT or PT), both of which have been previously shown to be significantly more effective in treating individuals with BPD than TAU [18, 119]. Thus, the comparison group in this study can be considered of high quality. Together with the study’s focus on investigating mechanisms of psychotherapeutic change, this enhances the study’s potential to shed increasing light on how to best provide help for individuals with BPD in the future.

In addition, the trial offers the unique opportunity to overcome methodological limitations of former trials by sufficient sample powering, use of valid measures as well as investigating cost-effectiveness, adverse events and side effects of treatment. MBT is a manualized treatment with feasible training and supervision demands. A broad national dissemination and with this potentially improve routine care of BPD patients in Germany represents a key ultimate goal of the study.

## Supplementary Information

Supplementary Material 1.

## Data Availability

No datasets were generated or analysed during the current study.

## Abbreviations

3RS: Rupture Resolution Rating System
AD-SUS: Adult Service Use Schedule
ATQ-R: Automatic Thoughts Questionnaire – Revised
BEFKI-GC-K: Berlin Test for the Assessment of Fluid and Crystallized Intelligence
BFT: Bona fide treatment
BMI: Body Mass Index
BPD: Borderline Personality Disorder
BSL- 23: Borderline Symptom List – Short Version
CAMSQ: Certainty About Mental States Questionnaire
CBT: Cognitive Behavioural Therapy
CEA: Cost-effectiveness analysis
CI: Coordinating Investigator
CPPS: Comparative Psychotherapy Process Scale
CPTSD: Complex Posttraumatic Stress Disorder
DALYs: Disability adjusted life years
DASS- 21: Depression, Anxiety and Stress Scale – 21 Items
DBT: Dialectic Behavioural Therapy
DERS: Difficulties in Emotion Regulation Scale
DSM- 5: Diagnostic and Statistical Manual of Mental Disorders, Fifth Edition
DSM-IV: Diagnostic and Statistical Manual of Mental Disorders, Fourth Edition
DSMB: Data Safety Monitoring Board
DSS- 4: Dissociation-Tension Scale- 4
ECR-RD8: Experiences in Close Relationships – Revised Screening Version
EMA: Ecological Momentary Assessment
ETMCQ: Epistemic Trust, Mistrust and Credulity Questionnaire
GQ: Group Questionnaire
GQ- 12: Group Questionnaire – Short Version
GQ-D: German version of the Group Questionnaire
ICD- 10: International Classification of Diseases, Version 10
ICD- 11: International Classification of Diseases, Version 11
ICER: Incremental cost-effectiveness ratio
IPDE: International Personality Disorder Examination
IQ: Intelligence Quotient
ITI: International Trauma Interview
ITQ: International Trauma Questionnaire
KDEF: Karolinska Directed Emotional Faces
LPFS-BF: Levels of Personality Functioning Scale – Brief Form 2.0
MAIA- 2: Multidimensional Assessment of Interoceptive Awareness, Version 2
MBT: Mentalization-based Treatment
MBT-ACS: Mentalization-Based Treatment Adherence and Competence Scale
MEQ: Mentalizing Emotions Questionnaire
NEQ: Negative Effects Questionnaire
NSSI: Non-suicidal self-injury
PACS: Patient Attachment Coding System
PID5BF+M: Personality Inventory for DSM- 5 and ICD- 11 – Brief Form Plus Modified
PT: Psychodynamic Psychotherapy
PTSD: Posttraumatic Stress Disorder
RCT: Randomized-controlled trial
RFQ: Reflective Functioning Questionnaire
RFQ- 8: 8-Item Reflective Functioning Questionnaire
RPM- 2: Raven’s Progressive Matrices 2, Clinical Edition
SAM: Self-Assessment Manikin
SAS: Statistical Analysis System
SCID- 5-CV: Clinical Version of the Structured Clinical Interview for DSM- 5 Disorders
SCM: Structured clinical management
SPQ: Social Pain Questionnaire
STAXI- 2: State-Trait Anger Expression Inventory – 2
STiP 5.1: Semi-Structured Interview for Personality Functioning DSM- 5
TAI: Therapeutic Agency Inventory
TASc: Therapist Attunement Scales
TAU: treatment-as-usual
WAI-SR: Working Alliance Inventory – revised short version
WHODAS 2.0: World Health Organization Disability Assessment Schedule 2.0 Short Form
WSAS: Work and Social Adjustment Scale
