# Supplementary material for: Mentalization-based treatment versus bona fide treatment for patients with borderline personality disorder in Germany (MAGNET): study protocol of a prospective, multi-centre randomized controlled trial
Source: BMC Psychiatry. 2025 Apr 11;25:367. doi: 10.1186/s12888-025-06809-0 (PMC11992892; doi:10.1186/s12888-025-06809-0)
Supplement: Supplementary file 1 — Supplementary Material 1. [file 12888_2025_6809_MOESM1_ESM.pdf]

UNIVERSITÄTS
KLINIKUM
HEIDELBERG

Institut für Psychosoziale Prävention | Bergheimer Straße 54 | 69115 Heidelberg

Informationsschrift für Patient:innen

zur Teilnahme an einer klinischen Studie

Prüfer:in: ______________________________

Name in Druckbuchstaben

Prüfstelle *(Stempel mit Telefonnummer)*

Titel:

Mentalisierungsbasierte Therapie versus Bona-fide-Therapie für Patient:innen mit Borderline-Persönlichkeitsstörung in Deutschland (MaGnet): eine prospektive, multizentrische randomisiert-kontrollierte Studie

Sehr geehrte:r Patient:in,

mit diesem Schreiben laden wir Sie ein, an der oben genannten Studie teilzunehmen. Bitte lesen Sie sich die folgenden Informationen sorgfältig durch. Sie können dann entscheiden, ob Sie teilnehmen möchten oder nicht. Lassen Sie sich ausreichend Zeit und stellen Sie den Studienmitarbeiter:innen alle Fragen, die für Sie wichtig sind. Die Studie wird finanziert durch die Deutsche Forschungsgemeinschaft (DFG). Die Studie wurde der zuständigen Ethikkommission vorgelegt. Sie hat keine Einwände erhoben. Die Studie wird in 5 Zentren in Deutschland durchgeführt und es sollten daran ca. 300 Patient:innen und ca. 100 Therapeut:innen teilnehmen.

Welches Ziel verfolgt die Studie?

In dieser Studie soll die Wirksamkeit der Mentalisierungsbasierten Therapie (MBT) bei der Behandlung von suizidalem und nicht-suizidalem selbstverletzendem Verhalten von Menschen mit Borderline Persönlichkeitsstörung (BPS) in Deutschland überprüft werden. Dafür soll die MBT verglichen werden mit zwei der vier Richtlinien-Psychotherapien in Deutschland, nämlich der Kognitiven Verhaltenstherapie (KVT) oder der Tiefenpsychologisch fundierten Psychotherapie (TfP). Die MBT wurde speziell für die Behandlung von Personen mit einer BPS entwickelt. Die MBT geht davon aus, dass Einschränkungen in der Mentalisierungsfähigkeit zur BPS beitragen. Mentalisieren ist die Fähigkeit, sich Verhalten auf Basis von vermuteten inneren, psychischen Zuständen zu erklären. Die empirische Forschung hat gezeigt, dass die Mentalisierungsfähigkeit bei Personen mit BPS besonders in Bezug auf emotional intensive Beziehungen Beeinträchtigungen aufweist. Die Wirksamkeit der MBT wurde international bereits in mehreren Studien belegt und soll nun auch in Deutschland überprüft werden mit dem übergeordneten Ziel der Verbesserung der Versorgung von Menschen mit einer BPS in Deutschland. Weiterhin soll untersucht werden, ob die MBT kosteneffektiver ist als die beiden Richtlinientherapien und effektiver in der Verbesserung der allgemeinen und interpersonellen Funktionsfähigkeit, BPS- und allgemeiner Symptomschwere, sozialer Anpassung, Lebensqualität, Reduktion der psychotropen Medikation und der Therapieteilnahme. Hierfür werden Daten zu Ihrem Wohlbefinden und Symptomen, Ihrer Therapie, Ihrer beruflichen Tätigkeit und ggf. Medikamenteneinnahme erfasst sowie diagnostische Termine und Therapiesitzungen auf Video aufgezeichnet. Die Aufnahmen dienen dazu, die Qualität der durchgeführten Therapie für Patient:innen mit einer Borderline-Persönlichkeitsstörung zu gewährleisten und die MBT weiterzuentwickeln.

Wie läuft die Studie ab?

Die Studienteilnahme dauert ungefähr zwei Jahre. Im ersten Jahr bekommen Sie eine Therapie und im zweiten Jahr folgt die Nachbeobachtungsphase. Zu Beginn der Studie sollen Sie einen Termin vor Ort im Studienzentrum wahrnehmen, der in etwa 5 Stunden dauert. Bei diesem Termin werden zur Überprüfung der Teilnahmekriterien eine psychologische Diagnostik im

Interview und ein Intelligenztest durchgeführt. Dieses Interview wird auf Video aufgenommen und ggf. für eine Überprüfung der Zuverlässigkeit der Diagnosestellung im Rahmen der Studie ausgewählt. Dies bedeutet, dass sich weitere Diagnostiker:innen der Studie das Video anschauen und ebenfalls eine Diagnose stellen. Dies dient der Qualitätssicherung der Studie und hat keine weiteren Auswirkungen auf Ihre Teilnahme an der Studie oder Therapie. Wenn Sie die Teilnahmekriterien erfüllen und teilnehmen möchten, werden Sie entweder der MBT oder einer der beiden Richtlinien-Psychotherapien zugewiesen. In diesem Fall erhält Ihr:e Therapeut:in die Ergebnisse des diagnostischen Interviews. Falls Ihre Therapie an einem Ausbildungsinstitut für Psychotherapie durchgeführt wird, erhält das Ausbildungsinstitut die Ergebnisse des diagnostischen Interviews. Falls Sie diese Daten dort für weitere Forschungszwecke zur Verfügung stellen möchten, werden Sie dort gebeten, eine Einwilligung zu erteilen.

Kurz vor dem Start Ihrer Therapie werden Sie darüber hinaus gebeten, online ausführlich Fragen zu Ihrem Befinden und Symptomen, Ihrer beruflichen Tätigkeit, Kosten, die mit Ihrer Erkrankung verbunden sind, und ggf. Medikamenteneinnahme zu beantworten. Außerdem sollen Sie eine Aufgabe am PC bearbeiten. Bei dieser Aufgabe werden Ihnen Gesichter mit unterschiedlichen Emotionen präsentiert, die Sie durch Drücken einer entsprechenden Taste so schnell wie möglich, je nach gezeigter Emotion, klassifizieren sollen. Die Bearbeitung der Online-Erhebung (Fragen plus PC-Aufgabe) dauert etwa 90 Minuten. Die Entscheidung, ob Sie MBT oder Richtlinien-Psychotherapie erhalten, wird über ein zuvor festgelegtes Zufallsverfahren getroffen, vergleichbar mit dem Werfen einer Münze im Verhältnis 1:1 (genannt eine Randomisierung). Dies geschieht sobald eine Gruppe von Patient:innen als geeignet zur Teilnahme identifiziert wurde und kann ggf. zu einer Wartezeit führen, bis Sie mit Ihrer Therapie starten können.

Im ersten Jahr der Studie erhalten Sie dann eine MBT oder eine Richtlinien-Psychotherapie (KVT oder TfP). Sollten Sie der Richtlinien-Psychotherapie zugeordnet werden, können Sie sich entscheiden, ob Sie eine KVT oder TfP beginnen möchten. Unabhängig davon, ob Sie MBT oder Richtlinien-Psychotherapie erhalten, werden Sie begleitend zur Therapie immer wieder gebeten, online auf Ihrem Smartphone oder am PC Fragen zu Ihrem Wohlbefinden und Symptomen, Ihrer Therapie, Ihrer beruflichen Tätigkeit und ggf. Medikamenteneinnahme zu

beantworten. Die Abfragen finden zu unterschiedlichen Zeitpunkten statt: Jeweils in einer Woche pro Monat erfolgen vier Abfragen (je im Abstand von 2 Tagen) zum Auftreten von Selbstverletzendem Verhalten und Ihrem momentanen Empfinden. Die Bearbeitungsdauer pro Abfrage beträgt ca. 5 Minuten (20 Minuten über die ganze Woche hinweg). Nach 6 und 12 Monaten (in der Mitte und am Ende der Therapie) werden Sie wie zu Beginn der Studie gebeten, ausführlichere Fragen zu Ihrem Befinden und der Therapie, Ihrer Berufstätigkeit und Kosten, die mit Ihrer Erkrankung verbunden sind, sowie ggf. zu Veränderungen in der Medikamenteneinnahme zu beantworten. Die Bearbeitungsdauer für diese ausführliche Abfrage beträgt in etwa 90 Minuten pro Messzeitpunkt. Nach jeder Therapiesitzung findet eine kurze Abfrage zur Therapie statt. Diese Abfrage dauert ca. 5 Minuten.

Auch Ihr:e Therapeut:in wird gebeten, im Rahmen der Studie wenige Angaben zu Ihrer Therapie zu machen: Der:die Therapeut:in wird gebeten, Datum und Anzahl der durchgeführten Therapiesitzungen und erhaltener Supervision (fachliche Beratung zur Therapie durch erfahrenere Therapeut:innen) anzugeben sowie ebenfalls die Qualität der therapeutischen Beziehung mit jeweils einem Fragebogen nach jeder Einzel- bzw. Gruppensitzung einzuschätzen.

Außerdem besteht zum Schutze der teilnehmenden Patient:innen in der Studie eine besondere Pflich, dass das Auftreten schwerwiegender Ereignisse schnellstmöglich an die Studienleitung gemeldet wird. Dafür werden Ihre Therapeut:innen und das Studienpersonal gebeten, die folgenden schwerwiegende Ereignissen an die Studienleitung zu melden, sobald sie von diesen erfahren, spätestens jedoch innerhalb von 7 Tagen.

Schwerwiegende Ereignisse sind in der Studie Ereignisse, die

- Tod zur Folge haben
- lebensbedrohlich sind (hierzu gehören alle Suizidversuche)
- einen Krankenhausaufenthalt notwendig machen oder diesen verlängern
- andauernde oder schwerwiegende Behinderung zur Folge haben

- wichtige Ereignisse, die den:die Patient:in gefährden oder ohne Intervention eins der o.g. Ereignisse zur Folge haben könnten (z.B. Krämpfe, Entwicklung einer Abhängigkeit)

Nach der Meldung wird der:die Therapeut:in oder die Person des Studienteams, der:die Meldung gemacht hat, gebeten, Informationen zum Ereignis (was geschehen ist, Dauer, in welcher Therapiephase sich der:die Patient:in befand) zu geben und einzuschätzen, ob das Ereignis durch die Studienteilnahme verursacht wurde. Auf Basis dieser Informationen schätzt die Studienleitung ebenfalls ein, ob das Ereignis durch die Studienteilnahme verursacht sein könnte. All diese Informationen werden an ein Data Safety Management Board weitergeleitet, die sich beraten und bei gehäuftem Auftreten schwerwiegender Ereignisse in der Studie empfehlen können, die Studie abzubrechen. Diese Entscheidung obliegt dann der Studienleitung.

Im zweiten Jahr der Studie (Nachbeobachtungphase) erfolgen die Abfragen etwas seltener: Die vier Abfragen in einer Woche mit je ca. 5 Minuten Bearbeitungsdauer zu Selbstverletzendem Verhalten finden dann noch alle drei Monate statt. 24 Monate nach Studienbeginn findet der letzte Studientermin statt. Dieser wird online im Interview mit einem:r Studienmitarbeiter:in durchgeführt und dauert in etwa 1,5 Stunden. Im Interview werden eine psychologische Diagnostik und eine Therapieevaluation durchgeführt und nach dem Interview sollen Sie nochmals die Aufgabe am PC bearbeiten, bei der Gesichter mit unterschiedlichen Emotionen präsentiert werden, die Sie richtig klassifizieren sollen. Auch hier werden Sie nochmals gebeten, zusätzlich für ca. 90 Minuten online ausführliche Fragen zu Ihrem Befinden und der Therapie, Ihrer Berufstätigkeit und Kosten, die mit Ihrer Erkrankung verbunden sind, sowie ggf. zu Veränderungen in der Medikamenteneinnahme zu beantworten.

Folgendes Schema veranschaulicht den Studienablauf:

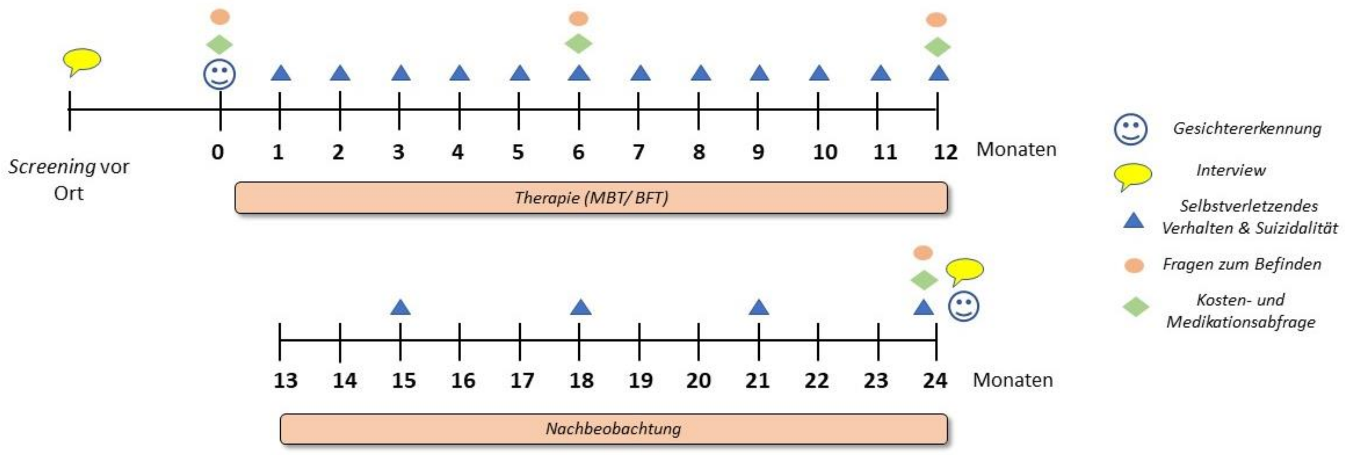

Abbildung 1: Ablauf der Studie und Zeitpunkte der unterschiedlichen Abfragen (exkl. kurze Abfragen im Anschluss an jede Therapiesitzung).

Wie läuft die Therapie ab?

Sollten Sie der Therapie „MBT“ zugeordnet werden, werden Sie durchschnittlich zwei Therapietermine pro Woche erhalten: ein Termin ist eine Gruppen-Sitzung mit anderen Studienteilnehmenden. Diese Gruppentermine werden durch zwei Therapeut:innen geleitet. Der zweite Termin ist eine Einzelsitzung mit Ihrer:m Psychotherapeut:in. Insgesamt finden so ca. 60 Therapiesitzungen statt. Dabei beinhalten 8-10 der Gruppensitzungen eine „Psychoedukation“, d.h. hier werden Informationen zur Borderline Persönlichkeitsstörung und möglichen Schwierigkeiten bei der Mentalisierungsfähigkeit vermittelt. Die Mentalisierungsfähigkeit beschreibt die Fähigkeit, sich psychische Gründe für das eigene Verhalten und das Verhalten anderer vorzustellen und für die eigene Regulation nutzen zu können. Die MBT zielt darauf ab, über ein Trainieren der Mentalisierungsfähigkeit eine Verbesserung in der Selbst- und Beziehungsregulation zu erreichen.

Sollten Sie der Richtlinien-Psychotherapie zugeordnet werden, erhalten Sie eine KVT oder TfP, die in der Regel einen Einzeltermin in der Woche bei Ihrer:m Psychotherapeut:in beinhaltet. Im Durchschnitt zeigen Ergebnisse, dass in diesen Therapien ca. 48 Termine stattfinden. Je nach Angebot Ihrer:s Psychotherapeut:in können ebenfalls zusätzliche Gruppensitzungen

erfolgen. KVT stützt sich auf die Lern- und Sozialpsychologie und zielt darauf ab, dass Patient:innen neues hilfreiches Verhalten erlernen, um mehr Selbstkontrolle und Wohlbefinden zu erlangen. Die TfP zielt darauf ab, über die Nutzung der therapeutischen Beziehung unbewusst wirksame, belastende Muster in zwischenmenschlichen Beziehungen sowie Schwierigkeiten in der Selbst- und Beziehungsregulation zu erforschen und bearbeiten.

Alle Therapiestunden (Einzel- und Gruppensitzungen) werden auf Video- bzw. Tonband aufgenommen. Dies dient einerseits der Qualitätssicherung der Behandlung, andererseits werden die Videos im Rahmen der Studie zu Forschungszwecken genutzt, um genauere Aussagen zu Wirksamkeit und Verlauf der Behandlung treffen zu können und somit die das Verständnis über Wirkmechanismen zu vertiefen.

Habe ich einen persönlichen Nutzen?

Wir können Ihnen nicht garantieren, dass Sie einen persönlichen Nutzen von der Teilnahme an der Studie haben werden. Durch die Teilnahme an der Studie unterstützen Sie die Forschung zur Borderline Persönlichkeitsstörung. Die Ergebnisse dieser Studie können dazu beitragen, dass für andere Patient:innen, die an Ihrer Erkrankung leiden, die Versorgung verbessert wird. Sie erhalten eine psychotherapeutische Behandlung im Rahmen der kassenärztlichen Versorgung, deren Qualität durch regelmäßige therapeutische Supervision in der Studie gesichert und durch wissenschaftliche Erhebungen begleitend untersucht wird.

Welche Risiken sind mit der Teilnahme verbunden?

Die Psychotherapie soll Ihnen dabei helfen, ein besseres Wohlbefinden zu erreichen. Durch die Beschäftigung mit schwierigen Themen können dabei zeitweise Verschlechterungen im Wohlbefinden auftreten, was für den Prozess notwendig sein kann. Es können jedoch auch Nebenwirkungen einer Psychotherapie wie eine dauerhafte Verschlechterung im Befinden auftreten. Solche Nebenwirkungen werden im Rahmen dieser Studie systematisch erfasst. Auch können die Fragebogenerhebungen als psychisch belastend empfunden werden. Sie können die Teilnahme an der Studie jederzeit abbrechen. In diesem Fall werden wir Sie fragen, ob Sie an einem Therapieevaluationsinterview teilnehmen möchten.

Entstehen mir durch die Teilnahme Kosten? / Erhalte ich eine Bezahlung bzw. Aufwandsentschädigung?

Die Studienteilnahme ist für Sie kostenlos. Sie erhalten eine Aufwandsentschädigung von insgesamt maximal 400 Euro für die planmäßige Bearbeitung der wissenschaftlichen Erhebungen: Für die Teilnahme am Einschlussinterview zu Beginn der Studie und Abschlussinterview nach 24 Monaten inklusive der Bearbeitung der dazugehörigen Fragebögen erhalten Sie je 100 €. Auch für die Teilnahme an den monatlichen bzw. dreimonatigen Abfragen zum Selbstverletzenden Verhalten und momentanem Empfinden erhalten Sie insgesamt 100 €. Je 50 € erhalten Sie für die Teilnahme an den ausführlichen Online-Abfragen in der Mitte (nach 6 Monaten) und am Ende der Therapie (nach 12 Monaten). Für etwaige mit der Wahrnehmung von dem ersten Studientermin vor Ort (nicht der Therapietermine) verbundene Fahrtkosten und Parkgebühren erhalten Sie ebenfalls eine angemessene Aufwandsentschädigung (bei Anfahrt mit dem eigenen Auto € 0,30/km, Erstattung von Parkgebühren bzw. des Fahrpreises für öffentliche Verkehrsmittel (bei der Deutschen Bahn nur zweite Klasse) gegen Vorlage geeigneter Belege. Über die Angabe Ihres Namens, Ihrer Adresse und IBAN wird Ihnen innerhalb von wenigen Wochen die Aufwandsentschädigung auf Ihr Konto ausgezahlt.
Wenn Sie aus Gründen aus der klinischen Studie ausscheiden, wird eine anteilige Aufwandsentschädigung gezahlt, entsprechend dem bisherigen Umfang der vorgenommenen Abfragen.

Bin ich während der klinischen Studie versichert?

Eine Wegeunfallversicherung ist für Sie abgeschlossen. Hier sind Sie gegen Schäden, die bei direkten An- und Abreisen zum ersten Studientermin auftreten könnten, versichert. Auch für diese Versicherung sind Sie verpflichtet, etwaige Schäden unverzüglich dem Versicherer zu melden, gegebenenfalls mit Unterstützung durch Ihre:n Studienärzt:in, um Ihren Versicherungsschutz nicht zu gefährden.

**Name und Anschrift der Versicherung:**	SV SparkassenVersicherung AG<br>Gebäudeversicherung AG<br>Bahnhofstraße 69, 65185 Wiesbaden
Telefon:	0611 178-100
Fax:	0611 178-109
Versicherungsnummer:	50102038053

Sie erhalten ein Exemplar der Versicherungsbestätigung einschließlich der Versicherungsbedingungen.

Welche anderen Therapien werden für die Behandlung der BPS empfohlen?

Für die Behandlung der BPS werden aktuell neben der MBT vor allem die Dialektisch Behaviorale Therapie (DBT), aber auch Schematherapie und Übertragungsfokussierte Therapie als evidenzbasierte Therapiemethoden empfohlen. Für die DBT gibt es dabei bislang am meisten Belege der Wirksamkeit, gefolgt von der MBT. Es gibt erste Hinweise darauf, dass die DBT besonders wirksam bei der Verbesserung des Schweregrades der Symptomatik, des Selbstverletzenden Verhaltens und des psychsozialen Funktionsniveaus ist, und die MBT ebenfalls bei der Verbesserung des Selbstverletzenden Verhaltens sowie bei Suizidalität. In der gängigen Praxis werden für die Behandlung von Menschen mit einer BPS außerdem häufig die vier Richtlinien-Therapien durchgeführt (Verhaltenstherapie, Tiefenpsychologisch fundierte Psychotherapie, analytische Psychotherapie und systemische Therapie). Die Wartezeit für eine Psychotherapie beträgt aktuell bundesweit im Schnitt etwa 5 Monate (Quelle: Deutscher Bundestag, 2022).

Wenn Sie eine dieser Behandlungen anstatt der Studienteilnahme wünschen, können Sie sich über die Studienmitarbeitenden oder z.B. auf der Website der Psychotherapeutenkammer über weitere mögliche Anlaufstellen/Therapeut:innen informieren: https://www.lpk-bw.de/psychotherapeutensuche

UNIVERSITÄTS
KLINIKUM
HEIDELBERG

Institut für Psychosoziale Prävention | Bergheimer Straße 54 | 69115 Heidelberg

Informationen zum Datenschutz

Allgemein:

Die ärztliche Schweigepflicht und datenschutzrechtliche Bestimmungen werden eingehalten. Rechtsgrundlage für die Datenverarbeitung ist Ihre freiwillige Einwilligung (Art. 6 Abs. 1 Buchst. c) DSGVO). Während der Studie werden medizinische Befunde und persönliche Informationen von Ihnen erhoben und in der Prüfstelle in Ihrer persönlichen Akte niedergeschrieben und elektronisch gespeichert. Die für die Studie wichtigen Daten werden zusätzlich mittels der Online-Plattform RedCap erhoben. Für jede Umfrage erhalten Sie einen über die Plattform automatisch versendeten Link per Email, über den Sie zur Umfrage gelangen. Für die automatische Versendung der Umfrage-Links und die Zuordnung der Daten zu den unterschiedlichen Messzeitpunkten zu Ihrer Person werden Ihre Emailadresse, Ihr Geburtsjahr sowie die Emailadresse Ihres:r Therapeut:in in die Datenbank eingetragen. RedCap ist eine sichere Web-basierte Anwendung zur Erfassung von Forschungsdaten, die von der Vanderbilt-Universität in Nashville, USA, entwickelt und herausgegeben wurde. Die Datenspeicherung erfolgt jedoch ausschließlich auf einem Server des Universitätsklinikums Heidelberg. Da es sich bei den Emailadressen und Ihrem Geburtsjahr um personenbezogene Daten handelt, d.h. Sie darüber identifizierbar sind, werden Ihre Daten vor unbefugtem Zugriff geschützt: Zugriff auf die Daten haben nur die Mitarbeiter:innen der Studie an der jeweiligen Prüfstelle und der Studienleitung sowie den Mitarbeitenden der Studienleitung. Eine Zuordnung Ihrer E-Mail-Adresse zu Ihren Daten ist somit nur diesen Studienmitarbeitenden möglich. Diese unterliegen einer absoluten Schweigepflicht. Die für die Studie wichtigen Daten werden in pseudonymisierter[1] Form noch für die Auswertung an das Koordinierungszentrum für Klinische Studien (KKS) der Medizinischen Fakultät des Universitätsklinikums Heidelberg weitergegeben. Für die Pseudonymisierung werden die personenbezogenen Daten durch einen Nummern-

[1] „Pseudonymisierung" ist die Verarbeitung personenbezogener Daten in einer Weise, dass die personenbezogenen Daten ohne Hinzuziehung zusätzlicher Informationen („Schlüssel") nicht mehr einer spezifischen betroffenen Person zugeordnet werden können. Diese zusätzlichen Informationen werden dabei gesondert aufbewahrt und unterliegen technischen und organisatorischen Maßnahmen, die gewährleisten, dass die personenbezogenen Daten nicht einer identifizierten oder identifizierbaren natürlichen Person zugewiesen werden.

und/oder Buchstabencode ersetzt. Zusätzlich werden ausgewählte Auswertungen Ihrer Daten ebenfalls in pseudonymisierter Form an die Mitglieder eines Sicherheitskommittees weitergegeben, wobei ein Mitglied seinen/ihren Sitz in der Schweiz hat. Die Studienleitung wird alle angemessenen Schritte unternehmen, um den Schutz Ihrer Daten gemäß den Datenschutzstandards der Europäischen Union zu gewährleisten. Die Daten sind gegen unbefugten Zugriff gesichert. Im Studienzentrum ist eine Liste hinterlegt, auf der die Namen den Nummern- und/oder Buchstabencodes zugeordnet sind. Diese Liste wird im Studienzentrum gesondert aufbewahrt und unterliegt dort technischen und organisatorischen Maßnahmen, die gewährleisten, dass die personenenbezogenen Daten Ihnen durch unbefugte Personen nicht zugeordnet werden können. Sie haben jederzeit das Recht Ihre Einwilligung zurückzuziehen und explizit den Wunsch zu äußern, die vollständige Löschung der von Ihnen erhobenen Daten zu veranlassen. Nach Beendigung der Datenerhebung wird die Emailadresse gelöscht. Ab diesem Zeitpunkt sind die Daten vollständig pseudonymisiert. Die erhobenen, pseudonymisierten Daten werden am Institut für Psychosoziale Prävention des Universitätsklinikums Heidelberg gespeichert, ausgewertet, 10 Jahre nach Erhebung aufbewahrt und anschließend vernichtet. Die Daten werden ausschließlich zu Zwecken dieser Studie verwendet. Die personenbezogenen Daten werden, so bald dies nach dem Forschungszweck möglich ist, anonymisiert. Bitte beachten Sie, dass ab diesem Zeitpunkt keine Löschung Ihrer Daten mehr verlangt werden kann.

Ihnen wird zur Auswahl gestellt, ob Sie für die Beantwortung der Fragebögen die MyCap App nutzen möchten. Die MyCap App ermöglicht die Datenerhebung über RedCap ohne Umfrage-Links direkt über die App auf Ihrem Handy. Wenn Sie die Studienumfragen ausschließlich über die App beantworten und keine technischen Probleme auftreten, ist die Eingabe Ihrer Emailadresse in RedCap nicht notwendig. Stattdessen installieren Sie die App auf Ihrem Handy und vergeben selbst einen frei wählbaren Profilnamen sowie eine 6-stellige PIN, die Sie zum Öffnen der App verwenden. Über einen individuellen QR-Code loggen Sie sich einmalig in das Studienprojekt ein und beantworten dann in der App die Umfragen. Die Eingabe der Emailadresse Ihrer Therapeut:in und Ihres Geburtsjahres wird dennoch in RedCap erfolgen, damit die Antworten Ihrer:Ihres Therapeut:in Ihrer Person zugeordnet werden können. MyCap ist eine frei verfügbare, auf die Teilnehmenden ausgerichtete

mobile Anwendung. Die App kann auf iOS- und Android-Geräten installiert werden. Alle Daten, die auf dem Gerät einer:eines Teilnehmenden erfasst werden, werden automatisch und sofort mit RedCap synchronisiert. Wenn Daten erfasst werden, während die Teilnehmenden offline sind, werden die Daten synchronisiert, sobald die Internetverbindung wiederhergestellt und die App geöffnet wird. Die folgenden Sicherheitsmaßnahmen wurden getroffen, um den Schutz der Daten zu gewährleisten: Die Daten der Teilnehmenden werden lokal auf dem Gerät in einer verschlüsselten Datenbank gespeichert. Die Daten bleiben auf dem Gerät, wenn keine Internetverbindung verfügbar ist. Dies gilt sowohl für iOS- (Apple) als auch für Android-Geräte. Wenn eine Internetverbindung verfügbar ist, werden die Daten verschlüsselt und direkt an RedCap übertragen. Die von den Teilnehmenden eingegebenen Daten werden nicht an anderer Stelle gespeichert oder an andere Stellen gesendet. Die Daten befinden sich auf dem Gerät der:des Teilnehmenden oder auf dem Server des Universitätsklinikums Heidelberg. Die Daten werden vom Gerät der:des Teilnehmenden gelöscht, nachdem die Mycap-App überprüft hat, dass die Daten erfolgreich übertragen worden sind.

Bitte beachten Sie, dass es bei technischen Fehlern dennoch notwendig sein kann, Ihre Emailadresse nach dem weiter oben beschriebenen Vorgehen in RedCap einzugeben, um Ihnen die Umfragen per Email zusenden und Ihrer Person zuordnen zu können.

Wenn Sie der zusätzlichen Verarbeitung Ihrer Daten für weitere Fragestellungen im Bereich psychische Gesundheit, Persönlichkeitsstörungen und Therapieprozessforschung zustimmen sowie zustimmen, dass wir Sie für weitere Studien kontaktieren dürfen, können Sie dies in der Einwilligungserklärung gesondert angeben.

Sie haben das Recht, von der:m Verantwortlichen (s.u.) Auskunft über die von Ihnen gespeicherten personenbezogenen Daten zu verlangen. Ebenfalls können Sie die Berichtigung unzutreffender Daten sowie die Löschung der Daten oder Einschränkung deren Verarbeitung verlangen. Wenn Sie von einem dieser Rechte Gebrauch machen wollen, wenden Sie sich bitte an Ihre:n Prüfer:in oder an der genanten Kontaktstelle.

Videoaufnahmen können nur unter sehr großem Aufwand vollständig pseudonymisiert werden. Diese Pseudonymisierung kann im Rahmen dieser Studie während der Aufnahmen nicht gewährleistet werden. Daher besteht die sehr geringe Wahrscheinlichkeit, dass eine an der Datenauswertung beteiligte Person den:die Teilnehmende:n in den Aufnahmen erkennt. Aus diesem Grund unterliegen alle an der Auswertung beteiligten Personen einer absoluten Schweigepflicht und dürfen unter keinen Umständen vertrauliche Informationen an Dritte weitergeben. Das Videomaterial wird mit der Software Veracrypt verschlüsselt auf Datenträgern zum Studienzentrum zur dortigen Datensicherung transportiert. Außerdem erfolgt der verschlüsselte Datentransport zu den jeweiligen Supervisor:innen der Therapien (erfahrene Therapeut:innen, die den oder die behandelnde Therapeut:in fachlich beraten). Hier werden sowohl der:die Supervisor:in, als auch die an der Supervision beteiligten Therapeut:innen einzelne Videos ansehen. Alle Beteiligten unterliegen der Schweigepflicht. Die Videoaufnahmen werden am Studienzentrum passwortgeschützten Computer aufbewahrt, der regelmäßig mit einem handelsüblichen Anti-Viren-Programm auf Viren geprüft wird. Das Videomaterial wird nur zu Forschungs- sowie Supervisionszwecken verwendet. Die Auswertung der Videoaufnahmen erfolgt unvollständig pseudonymisiert, d. h. unter Verwendung einer Nummer und ohne Angabe des Namens des:der o.g. Teilnehmenden. Es existiert eine Kodierliste auf Papier, die den Namen des:der o.g. Teilnehmenden mit der Nummer verbindet.

Das Videomaterial sowie alle Kopien werden nach Abschluss der Auswertung und Transkription direkt gelöscht, spätestens am 31.12.38. Beachten Sie, dass wegen der Videoaufnahmen eine Anonymität dieser Daten grundsätzlich nicht zugesichert werden kann.

Die Verantwortliche für die studienbedingte Erhebung personenbezogener Daten ist:

Prof. Dr. phil. Svenja Taubner
Direktorin des Instituts für Psychosoziale Prävention des Universitätsklinikums Heidelberg
Bergheimer Str. 54, 69115 Heidelberg
Email: Svenja.Taubner@med.uni-heidelberg.de
Tel: 06221-56 4701

Sind mit der Datenverarbeitung Risiken verbunden?

Bei jeder Erhebung, Speicherung, Nutzung und Übermittlung von Daten bestehen Vertraulichkeitsrisiken (z.B. die Möglichkeit, die betreffende Person zu identifizieren). Diese Risiken lassen sich nicht völlig ausschließen und steigen, je mehr Daten miteinander verknüpft werden können. Die Initiatorin der Studie versichert Ihnen, alles nach dem Stand der Technik Mögliche zum Schutz Ihrer Privatsphäre zu tun und Daten nur an Stellen weiterzugeben, die ein geeignetes Datenschutzkonzept vorweisen können. Medizinische Risiken sind mit der Datenverarbeitung nicht verbunden.

Im Rahmen dieser Studie erfolgt eine Weitergabe Ihrer pseudonymisierten Daten zum Zweck der Datenauswertung und Überwachung an andere Forscher:innen auch außerhalb der Europäischen Union und des Europäischen Wirtschaftsraumes (Schweiz). Mit Ihrer Einwilligung stimmen Sie zu, dass die Daten auch in diese Länder übermittelt werden dürfen.

Bei Anliegen zur Datenverarbeitung und zur Einhaltung der datenschutzrechtlichen Anforderungen können Sie sich an folgenden Datenschutzbeauftragten Ihrer Einrichtung wenden:

Datenschutzbeauftragter des Prüfzentrums
Datenschutzbeauftragter des Universitätsklinikum Heidelberg
Im Neuenheimer Feld 672
69120 Heidelberg
Tel.: 06221 56-7036
E-Mail: Datenschutz@med.uni-heidelberg.de

Datenschutzbeauftragter des Sponsors
Datenschutzbeauftragter des Universitätsklinikum Heidelberg
Im Neuenheimer Feld 672
69120 Heidelberg
Tel.: 06221 56-7036
E-Mail: Datenschutz@med.uni-heidelberg.de

Im Falle einer rechtswidrigen Datenverarbeitung haben Sie das Recht, sich bei folgender Aufsichtsbehörde zu beschweren:

Für den Sponsor bzw. dessen Vertreter innerhalb der EU zuständige Datenschutzaufsichtsbehörde:

Der Landesbeauftragte für den Datenschutz und die Informationsfreiheit Baden-Württemberg
Postfach 10 29 32, 70025 Stuttgart
Lautenschlagerstraße 20, 70173 Stuttgart
Tel.: 0711/61 55 41 – 0
Fax: 0711/61 55 41 – 15
E-Mail: poststelle@lfdi.bwl.de
Internet: http://www.baden-wuerttemberg.datenschutz.de

Sie haben ein Beschwerderecht bei jeder Aufsichtsbehörde für den Datenschutz. Eine Liste der Aufsichtsbehörden in Deutschland finden Sie unter:

https://www.bfdi.bund.de/DE/Infothek/Anschriften_Links/anschriften_links-node.html

Freiwilligkeit / Rücktritt

Die Teilnahme an der Studie erfolgt freiwillig. Falls Sie teilnehmen möchten, bitten wir Sie, die beiliegende Einwilligungserklärung zu unterschreiben. Sie können diese Einwilligung jederzeit schriftlich oder mündlich ohne Angabe von Gründen widerrufen, ohne dass Ihnen dadurch Nachteile entstehen. Wenn Sie Ihre Einwilligung widerrufen möchten, wenden Sie sich bitte an die Studienleitung oder das Sie behandelnde Personal. Wenn Sie Ihre Einwilligung widerrufen, werden keine weiteren Daten mehr erhoben. Bei einem Widerruf können Sie entscheiden, ob die von Ihnen studienbedingt erhobenen Daten gelöscht und die von Ihnen erstellten Aufnahmen (mit Ausnahme der Aufnahmen in der Therapie-Gruppe) vernichtet werden sollen oder weiterhin für die Zwecke der Studie verwendet werden dürfen. Auch wenn Sie einer weiteren Verwendung zunächst zustimmen, können Sie nachträglich Ihre Meinung

noch ändern und die Löschung der Daten und Vernichtung der Aufnahmen verlangen; wenden Sie sich dafür bitte ebenfalls an die Studienleitung oder das Sie behandelnde Personal. Beachten Sie, dass Daten, die bereits in wissenschaftliche Auswertungen eingeflossen sind nicht mehr auf Ihren Wunsch gelöscht werden können.
Falls Sie sich im Laufe der Studie entscheiden, Ihre Einwilligung zu ausschließlich den Video-/ Audioaufnahmen zurückzuziehen, dürfen Sie weiterhin an der Studie teilnehmen. Die in der Therapie-Gruppe erstellten Videoaufnahmen werden jedoch nicht vernichtet. Auch wird die Therapie-Gruppe weiterhin auf Video aufgenommen. Es erfolgt jedoch nach Aufnahme eine Anonymisierung[2] Ihrer Person durch Verpixelung, d.h. Ihre Gestalt wird auf einige farbige Blöcke reduziert und somit unkenntlich gemacht. Die Tonaufnahmen werden durch Verfälschung Ihrer Stimme unverständlich gemacht.

Weitere Informationen

Für weitere Informationen sowie für Auskünfte über allgemeine Ergebnisse und den Ausgang der Studie steht Ihnen als Koordinatorin der Studie Frau Dr. phil. Sophie Hauschild (Telefon: 06221-56 5662, Email: sophie.hauschild@med.uni-heidelberg.de) zur Verfügung.

Für Ihre Teilnahme an diesem Forschungsprojekt wären wir Ihnen dankbar!

[2] „Anonymisierung“ ist das Verändern personenbezogener Daten in der Weise, dass die betroffene Person nicht mehr oder nur mit einem unverhältnismäßig großen Kosten- oder Zeitaufwand identifiziert werden kann.

UNIVERSITÄTS
KLINIKUM
HEIDELBERG

Institut für Psychosoziale Prävention | Bergheimer Straße 54 | 69115 Heidelberg

Einwilligungserklärung zur Teilnahme an der klinischen Studie

Prüfer:in: ____
Name in Druckbuchstaben

**Prüfstelle** *(Stempel mit Telefonnummer)*

Titel:

Mentalisierungsbasierte Therapie versus Bona-fide-Therapie für Patient:innen mit Borderline-Persönlichkeitsstörung in Deutschland (MAGNET): eine prospektive, multizentrische randomisiert- kontrollierte Studie

....

Vor- und Nachname der Teilnehmer:in

geb. am ....

Ich habe die Informationsschrift gelesen und wurde zudem mündlich durch Herrn/Frau ____ über das Ziel und den Ablauf der Studie sowie über die Risiken ausführlich und verständlich aufgeklärt. Im Rahmen des Aufklärungsgesprächs hatte ich die Gelegenheit, Fragen zu stellen. Alle meine Fragen

wurden zu meiner Zufriedenheit beantwortet. Ich stimme der Teilnahme an der Studie freiwillig zu. Für meine Entscheidung hatte ich ausreichend Zeit. Ein Exemplar der Informationsschrift, der Einwilligungserklärung, sowie der Versicherungsunterlagen habe ich erhalten. Ein Exemplar vebleibt im Prüfzentrum.

Datenschutz

Mir ist bekannt, dass bei dieser Studie personenbezogene Daten verarbeitet werden sollen. Die Verarbeitung der Daten erfolgt nach gesetzlichen Bestimmungen und setzt gemäß Art. 6 Abs. 1 lit. a der Datenschutz-Grundverordnung folgende Einwilligungs-erklärung voraus:

Ich wurde darüber aufgeklärt und stimme freiwillig zu, dass meine in der Studie erhobenen Daten, insbesondere Angaben über meine Gesundheit[3] zu den in der Informationsschrift beschriebenen Zwecken in meiner persönlichen Akte an der Prüfstelle sowie über die Online-Plattform RedCap aufgezeichnet und in pseudonymisierter Form ausgewertet werden können. Ich stimme zu, dass an der Studie beteiligte Projektmitarbeitende Einblick in die von mir in der Studie gespeicherten Daten erhalten. Ich stimme zu, dass meine Therapeut:innen oder Studienpersonal schwerwiegende Ereignisse, die mich betreffen, in der beschriebenen Form weitergeben darf.

Ich bin darüber aufgeklärt worden, dass meine pseudonymisierten Daten auch in Drittländer (Schweiz) und an Empfänger weitergegeben werden könnten, für die kein Angemessenheitsbeschluss der Europäischen Kommission und auch keine anderen, gleichwertigen Datenschutzgarantien vorliegen. Ich bin darüber aufgeklärt worden, dass ich ohne meine Einwilligung in die Weitergabe meiner Daten in diese Länder nicht an dieser Studie teilnehmen kann.

[3] Gemäß Art. 9 Abs. 1 DSGVO handelt es sich bei Gesundheitsdaten um personenbezogene Daten besonderer Kategorie in deren Verarbeitung der:die Studienteilnehmer:in ausdrücklich einwilligen muss. Gleiches gilt für Daten, aus denen die rassische und ethnische Herkunft , politische Meinungen, religiöse oder weltanschauliche Überzeugungen oder die Gewerkschaftszugehörigkeit hervorgehen, sowie für die Verarbeitung von genetischen Daten, biometrischen Daten zur eindeutigen Identifizierung einer natürlichen Person, Daten zum Sexualleben oder zur sexuellen Orientierung.

Ich willige ausdrücklich in eine Weitergabe meiner Daten in Länder (Schweiz) außerhalb der Europäischen Union und des Europäischen Wirtschaftsraumes ein, in denen ein Schutz meiner Daten nicht in vergleichbarer Weise garantiert werden kann. Der erheblichen persönlichen Nachteile, die eine solche Datenübermittlung mit sich bringen kann, bin ich mir bewusst.

Ich willige ausdrücklich ein, dass die Ergebnisse meines diagnostischen Interviews an meine:n behandelnde:n Therpauet:in und ggf. an das Psychotherapieausbildungsinstitut weitergegeben werden, an dem meine Therapie stattfindet.

Ich wurde darüber aufgeklärt und stimme freiwillig zu, dass von mir und meinen Therapiesitzungen Video- bzw. Tonaufnahmen gemacht werden. Die Auswertung der Videoaufnahmen erfolgt unvollständig pseudonymisiert, d. h. unter Verwendung einer Nummer und ohne Angabe des Namens des:der o.g. Teilnehmenden. Die Einverständniserklärung für die Videoaufnahme ist freiwillig. Ohne diese ist eine Aufnahme in die Studie allerdings nicht möglich. Ich kann diese Erklärung jederzeit widerrufen. Im Falle einer Ablehnung oder eines Rücktritts im Laufe der Studie entstehen für mich keinerlei Kosten oder anderweitige Nachteile; meine Teilnahme an der Studie kann fortgesetzt werden, wobei nach der Aufnahme eine Verpixelung meiner Person durchgeführt wird. Die Videoaufnahmen der Therapie-Gruppe werden nicht vernichtet und fortgeführt.

Dritte erhalten keinen Einblick in personenbezogene Unterlagen. Bei der Veröffentlichung von Ergebnissen der Studie wird mein Name ebenfalls nicht genannt. Die Daten werden nach Studienabschluss 10 Jahre aufbewahrt und dann vernichtet. Mir ist bekannt, dass diese Einwilligung jederzeit schriftlich oder mündlich ohne Angabe von Gründen widerrufen werden kann, ohne dass mir dadurch Nachteile entstehen. Die Rechtmäßigkeit der bis zum Widerruf erfolgten Datenverarbeitung wird davon nicht berührt. In diesem Fall kann ich entscheiden, ob die von mir erhobenen Daten gelöscht werden sollen oder weiterhin für die Zwecke der Studie verwendet werden dürfen.

Ich stimme zu, dass mein:e Therapeut:in im Rahmen der Studie zu meiner Therapie Angaben über die durchgeführte Sitzungsanzahl macht und seine:ihre Einschätzungen zur Qualität der therapeutischen Beziehung abgibt.

Ich möchte die Verwendung meiner Daten für andere/künftige Forschungszwecke wie folgt zulassen:

☐**Psychische Gesundheit**

☐**Persönlichkeitsstörungen**

☐**Therapieprozessforschung**

☐**Kontaktaufnahme für die Anfrage zur Teilnahme an weiteren Studien**

Teilnehmer:in:

....
Vor- und Nachname der Teilnehmer:innen in **Druckbuchstaben** (eigenhändig eintragen)

....
Ort, Datum **(eigenhändig eintragen)**

....
Unterschrift der Teilnehmer:innen

Aufklärende Person

Die teilnehmende Person wurde von mir im Rahmen eines Gesprächs über das Ziel und den Ablauf der Studie sowie über die Risiken aufgeklärt. Ein Exemplar der Informationsschrift und der Einwilligungserklärung habe ich der teilnehmenden Person ausgehändigt.

....
Vor- und Nachname des informierenden Prüfarztes / der Prüfärztin (des Psychologen/ der Psychologin) in Druckbuchstaben

....
Ort, Datum **(eigenhändig eintragen)**

....
Unterschrift des informierenden Prüfarztes / der Prüfärztin (Psychologen/ Psychologin)
